# A Radiocarbon‐Based Framework to Assess Soil Organic Carbon Persistence and Vulnerability Across Land‐Use Types

**DOI:** 10.1111/gcb.70799

**Published:** 2026-03-17

**Authors:** Luisa I. Minich, Jeffrey Beem‐Miller, Benedict V. A. Mittelbach, Dylan Geissbühler, Annegret Udke, Daniel Wasner, Margaux Moreno Duborgel, Ciriaco McMackin, Alexander S. Brunmayr, Lukas Wacker, Philip Gautschi, Negar Haghipour, Markus Egli, Jens Leifeld, Timothy I. Eglinton, Frank Hagedorn

**Affiliations:** ^1^ Swiss Federal Institute for Forest, Snow and Landscape Research (WSL) Birmensdorf Switzerland; ^2^ Geological Institute, Department of Earth and Planetary Sciences ETH Zurich Zurich Switzerland; ^3^ Institute for Global Change Biology University of Michigan Ann Arbor MI USA; ^4^ Climate and Ecosystem Sciences Division Lawrence Berkeley National Laboratory Berkeley CA USA; ^5^ Laboratory for the Analysis of Radiocarbon With AMS, Department of Chemistry, Biochemistry and Pharmaceutical Sciences University of Bern Bern Switzerland; ^6^ Geochronology, Department of Geography University of Zurich Zurich Switzerland; ^7^ Soil Biogeochemistry Laboratory EPFL Sion Sion Switzerland; ^8^ Soil Resources, Department of Environmental Systems Science ETH Zurich Zurich Switzerland; ^9^ Laboratory for Ion Beam Physics, Department of Physics ETH Zurich Zurich Switzerland; ^10^ Climate and Agriculture Group Agroscope Zurich Switzerland

**Keywords:** biological stability, land‐use type, radiocarbon, SOC dynamics, SOC persistence, SOC stabilization, SOC vulnerability, system age, thermal stability, transit time

## Abstract

Soil organic carbon (SOC) can persist from days to millennia but remains vulnerable to carbon (C) loss upon disturbances, depending on environmental conditions and mode of stabilization. Understanding drivers of persistence and vulnerability is crucial to assess soil C sequestration as well as potential SOC losses due to changes in climate and land use. Here, we investigate SOC persistence and vulnerability in five land‐use types by combining radiocarbon‐derived estimates of SOC age (system age) and age of respired CO_2_ (transit time) with indicators of biological (SOC decomposability) and thermal stability (residual oxidisable carbon content, ROC). Based on this, we developed a vulnerability index for SOC and applied it across soil profiles from 19 sites representing temperate and alpine grasslands, forests, croplands, and managed peatlands. Transit times and system ages ranged from 2 years in the organic layer of forests to 5760 years in subsoils of managed peatlands and varied significantly across land‐use types and soil depth. Transit times were generally shorter than system ages, indicating that soil‐respired CO_2_ is dominated by more recent inputs, while bulk SOC contains more persistent C. In forests, temperate grasslands, and croplands, system ages were positively linked to thermal stability and mineral reactivity, indicating higher SOC persistence through organo‐mineral stabilization. In contrast, alpine grasslands and managed peatlands showed centennial to millennial system ages despite low thermal stability (< 10%‐ROC), reflecting inhibited microbial decomposition due to cold and/or anaerobic conditions in these ecosystems. In combination with high SOC stocks (> 90 kg m^−2^ in managed peatlands), this implies a high vulnerability of these soils to environmental disturbances that alleviate these constraints. Our findings demonstrate that combining metrics of biological and thermal stability with radiocarbon data provides a powerful framework to assess SOC vulnerability to disturbances induced by environmental change.

## Graphical abstract

Soil organic carbon (SOC) persists over timescales from years to millennia but remains vulnerable to disturbance depending on stabilization mechanisms and environmental constraints. This study assesses SOC persistence and vulnerability across five land‐use types by combining radiocarbon‐derived system age and transit time with indicators of biological and thermal stability. Across 19 sites, SOC persistence was linked to organo‐mineral stabilization in forests, temperate grasslands, and croplands. In alpine grasslands and managed peatlands, cold or anaerobic conditions preserved old but thermally labile SOC. These contrasts reveal high vulnerability where environmental constraints on decomposition may be alleviated by changes in climate or land use.

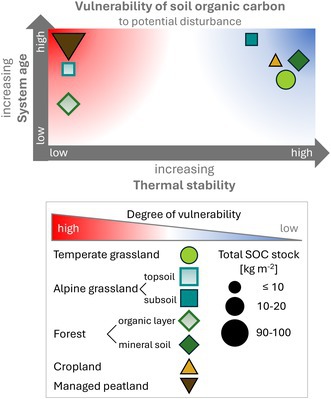

## 1 Introduction

Soils are a major pool in the global carbon (C) cycle, storing an estimated ~1700 Gt C—approximately twice the amount of C currently present in the atmosphere (~890 Gt C) (Friedlingstein et al. 2025). Microbial decomposition of soil organic carbon (SOC) releases large amounts of CO_2_ (~60 Gt C per year; Nissan et al. 2023). The time SOC persists in soils until it is released back to the atmosphere as CO_2_ via microbial decomposition can vary from days to millennia (Schmidt et al. 2011; Trumbore 2009). Generally, microbially respired CO_2_ is dominated by recent C, indicating that most new C inputs reside in soils for a relatively short time (Xiao et al. 2022) as microbes preferentially mineralize fresh substrates. In comparison, bulk SOC is a heterogeneous mixture of various C pools which differ in their age and persistence: plant‐derived particulate organic matter (POM) is generally younger and less persistent than the more microbially derived mineral‐associated organic matter (MAOM) (Heckman et al. 2022; Moreno‐Duborgel et al. 2025). In sum, these pools, which follow different dynamics and respond to distinct drivers, define the age and persistence of bulk SOC. Understanding SOC cycling and persistence is crucial to predict the capacity of soils to sequester C or to lose C and to assess how environmental conditions influence the balance between C storage and release.

Land use affects SOC cycling and persistence mainly through vegetation type and management practices (Oertel et al. 2016; Xiao et al. 2021). Vegetation affects the quantity (Schulze et al. 2009), quality (Liu et al. 2023; Zhang et al. 2008), and vertical distribution of C inputs to the soil (Jackson et al. 1996), which affects the partitioning of C inputs between above‐ and belowground pathways (Jackson et al. 1996) and the formation of POM and MAOM (Cotrufo et al. 2019; Sokol et al. 2022). In grasslands, high belowground C allocation, including easily degradable rhizodeposits (Fuchslueger et al. 2016; Hagedorn and Joos 2014; Wang et al. 2021), contributes to rapid decomposition of SOC (Finzi et al. 2015), but also enhances MAOM formation, promoting long‐term SOC stabilization (Sokol and Bradford 2019). In contrast, forest ecosystems, dominated by aboveground woody litter and recalcitrant C inputs with high lignin and polyphenol contents, exhibit slower decomposition, leading to the accumulation of organic layers and POM, primarily in upper soil layers (Cotrufo et al. 2015; Hiltbrunner et al. 2013; Zanella et al. 2011). The conversion of natural ecosystems to agricultural land use often results in substantial SOC loss (Emde et al. 2024; Poeplau and Don 2013). In croplands, intensive management—including biomass removal, tillage, and fertilization—reduces C inputs and disrupts the protection of SOC within soil structures, thereby decreasing SOC stocks (Keel et al. 2019) and altering the persistence of soil fractions (Heckman et al. 2022). In particular, the use of natural peatlands for agriculture can lead to substantial SOC loss due to drainage and subsequent oxidation of soil organic matter (SOM), releasing previously stored C back into the atmosphere (Leifeld et al. 2019; Wüst‐Galley et al. 2020).

Environmental disturbances, such as land‐use change and increasing temperatures, can destabilize weakly stabilized SOC by enhancing microbial decomposition (Hicks Pries et al. 2017; Poeplau and Don 2013; Soong et al. 2021). The degree to which SOC is vulnerable to environmental change across different land‐use types depends on the balance between SOC that is readily available for microbial decomposition and more stable SOC that is physically or chemically protected from microbial processing. The stability of SOC can be investigated by various measures. A widely used indicator of biological stability is SOC decomposability, defined as the amount of CO_2_ released under controlled conditions, normalized to the SOC content (Bader et al. 2017; Schädel et al. 2019). It reflects the proportion of SOC that is readily accessible to microbial activity. Another frequently used indicator is the thermal stability of SOC, based on the finding that thermally more stable fractions, which require more activation energy for oxidation, generally exhibit greater persistence in soil (Grant et al. 2019; Hemingway et al. 2019; Rennert and Herrmann 2022; Stoner, Schrumpf, et al. 2023). A thermally stable SOC fraction, referred to as residual oxidisable carbon (ROC) may comprise a variety of plant‐ and microbially‐derived compounds (e.g., De La Rosa et al. 2008; Giannetta et al. 2018; Stoner, Trumbore, et al. 2023) and can be quantified using a simple metric based on the oxidisable portion between 400°C and 600°C (Rennert and Herrmann 2022). In this study, we used both SOC decomposability and the proportion of ROC in total SOC as indicators of SOC stability.

Radiocarbon (^14^C) analysis is a powerful tool to investigate SOC dynamics, as it provides insight into the timescales over which C persists in soil (Trumbore 2009). ^14^C can be measured in both microbially respired CO_2_ and bulk SOC. When combined with soil C models, these measurements help to place constraints on SOC dynamics and enable estimates of transit time and system age, respectively (e.g., Beem‐Miller 2023; Shi et al. 2020; Tangarife‐Escobar et al. 2024; Xiao et al. 2022). Transit time is the age of the output flux and is defined as the time it takes for a C atom to transit the terrestrial ecosystem: from entering the ecosystem via photosynthetic C fixation, until leaving the soil through soil respiration (Sierra et al. 2017, 2018). System age is simply defined as the average age of SOC in bulk soil (Sierra et al. 2017) and is useful for characterizing SOC persistence (Sierra et al. 2018). The relationship between transit time and system age provides insights into SOC homogeneity: similar age metrics suggest that SOC is well mixed within the soil system and that all SOC has a comparable probability of being decomposed (Sierra et al. 2018). The degree of SOC homogeneity offers valuable insights into C cycling pathways within soils. For example, a pronounced difference between transit time and system age indicates that recent C inputs are rapidly respired, while only a small fraction contributes to the long‐term SOC pool (Xiao et al. 2022). In conjunction with the overall SOC stability, as defined by its thermal properties and decomposability, SOC homogeneity can serve as an indicator of a system's resilience to disturbance. For example, high SOC homogeneity combined with a dominance of weakly stabilized SOC indicates low resilience, as a large, uniformly vulnerable SOC pool is more susceptible to loss under disturbance.

**FIGURE 1:**
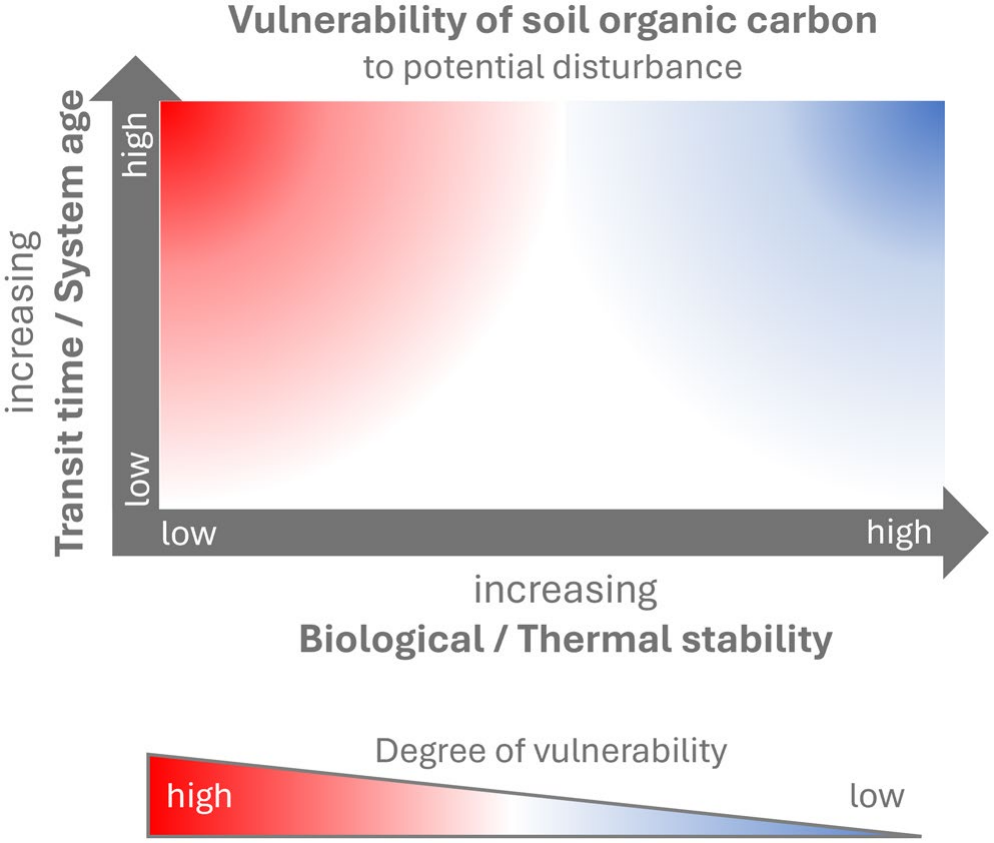
Conceptual figure illustrating indices of SOC vulnerability to potential disturbances caused by environmental change.

In our study, we combined transit times and system ages with indicators of biological and thermal stability to assess SOC persistence and to infer the vulnerability of SOC to disturbances induced by environmental changes in climate and land use (Figure 1). We assume that in soils characterized by high biological or thermal stability of SOC, along with longer transit times and older system ages, the high SOC persistence is primarily related to physicochemical protection from microbial decomposition. Due to its stabilization, SOC in these soils is less vulnerable to disturbances caused by environmental change. Conversely, in soils with low biological and thermal stability combined with longer transit times and older system ages, SOC must have persisted in soil despite being potentially available for microbial decomposition. This suggests that microbial decomposition of SOC in these soils is not primarily low due to physicochemical stabilization (which would enhance the biological and thermal stability), but rather due to suppressed microbial activity driven by environmental constraints such as low temperatures or limited oxygen availability. As a result, this old yet weakly stabilized SOC is highly vulnerable to decomposition under changing environmental conditions.

To assess SOC vulnerability, we applied a conceptual framework based on ^14^C‐derived ages and biological as well as thermal stability (Figure 1) across five dominant land‐use types in Switzerland—temperate and alpine grasslands, forests, croplands, and managed peatlands—and across soil depths down to 90 cm. We hypothesize that in land‐use types where SOC persistence is primarily governed by the inhibition of microbial activity due to environmental constraints, such as low temperatures or anaerobic conditions, SOC is more vulnerable to disturbances driven by environmental change. In contrast, in ecosystems where SOC is stabilized through mineral associations, we expect SOC to be less vulnerable to such disturbances. We further hypothesize that land‐use types resulting from past disturbances—such as the conversion of natural ecosystems to croplands or managed peatlands—have already undergone substantial losses of labile SOC. As a result, the remaining SOC pool reflects a higher degree of persistence, dominated by more stable fractions.

## 2 Materials and Methods

### 2.1 Study Sites

Soil was sampled at 19 sites from five dominant land‐use types across Switzerland: temperate grasslands (*n* = 3), alpine grasslands (*n* = 4), forests (*n* = 6), croplands (*n* = 3), and managed peatlands (drained and used for crop production; *n* = 3). Land‐use types were selected based on Land Use, Land‐Use Change, and Forestry (LULUCF) categories used by the IPCC (IPCC 2021, 2003) with additional differentiation between temperate and alpine grasslands to capture climatic variation. The sites vary in their soil physicochemical properties and cover a climatic gradient, with mean annual temperatures (MAT) ranging from −1.2°C to 10.7°C (Table S1). The site locations encompass Switzerland's five ecoregions (Figure S1) and represent the typical distribution of the respective land‐use type across Switzerland, reflecting typical climatic conditions and soil properties for their occurrence.

Grasslands were managed either as meadows (Chamau, Muldain), with practices including mowing and manure application, or as pastures (Jaun, alpine grasslands), used for grazing. Four of the six forest sites (Beatenberg, Lausanne, Novaggio, Vordemwald) are part of the Long‐term Forest Ecosystem Research programme LWF (Schaub et al. 2025) which belongs to the International Co‐operative Programme on Assessment and Monitoring of Air Pollution Effects on Forests (ICP forests, Ferretti and Fischer 2013; Sanders et al. 2016). The three cropland sites exhibit similar management practices and are part of long‐term field trials conducted by the Swiss Federal Research Institute Agroscope, Switzerland (for Changins: e.g., Maltas et al. 2018; Altwi and Reckenholz: e.g., Hirte et al. 2021). The agricultural field trials at the cropland sites were established over 35 years ago and had already been used as cropland before (Hirte et al. 2021; Maltas et al. 2018). All cropland sites were treated with mineral fertilizers following the Swiss fertilization guidelines (Flisch et al. 2009), were not actively limed, and were regularly ploughed to a depth of ~25 cm prior to sowing. The managed peatlands were drained in the second half of the 19th century and subsequently used for crop production since then (Leifeld et al. 2011).

### 2.2 Soil Sampling and Analysis

Apart from LWF forest and alpine grassland sites, all sites were sampled in summer 2023. For this, three soil cores were collected using a Humax corer (Ø = 5 cm) to a depth of 52 cm. Sampling was extended as deep as possible (maximum depth = 90 cm) using a soil auger (Ø = 5 cm). Soil samples were stored at 2°C until further processing. Mineral soil samples from the three soil cores were divided into depth intervals of 0–5, 5–10, 10–20, 20–40, and 40–90 cm at all sites. At forest sites, organic layers were additionally sampled, including the litter (Oi), moderately decomposed (Oe), and humified (Oa) horizons. Alpine grassland sites were sampled separately in summer 2022 by excavating soil profiles and collecting volume‐proportional samples for the predefined depth intervals. For further analysis, soil from replicated cores at corresponding depths was pooled and homogenized to one composite sample per depth and site. To assess CO_2_ release from heterotrophic respiration during incubation, a subsample of fresh soil was sieved to 4 mm, with roots and skeleton removed by hand. Another subsample was dried at 40°C and sieved to 2 mm. Dried samples were ground to a fine powder using a ball mill (MM2000, Retsch GmbH, Germany). At each LWF forest site, 16 replicated soil profiles were sampled across a 43 × 43 m area according to Walthert et al. 2002 in summer 2022. For each profile, a 0.5 × 0.5 m square was sampled for the organic layer and pre‐defined depth intervals (as mentioned above). The samples of the replicated plots were mixed and homogenized to four composite samples in the field. After drying, sieving (2 mm), removing roots and skeleton, as well as milling, the four replicated composite samples were further homogenized and mixed to one final composite sample per site and depth. Fine earth masses (bulk soil mass < 2 mm, excluding roots and skeleton) per m^2^ were used to calculate SOC stocks for all land uses.

Soil pH was determined potentiometrically for dried soil samples in a 0.01 M calcium chloride (CaCl_2_) solution at a ratio of 1:5 for mineral soil horizons and 1:20 for organic layers following an equilibration period of 120 min. Soil texture was measured by sedimentation after removing organic matter by treatment with hydrogen peroxide (Gee and Bauder 2018; Walthert et al. 2002), separating sand (0.063–2 mm), silt (0.002–0.063 mm), and clay (< 0.002 mm). Texture was not measured for managed peatlands dominated by organic matter.

### 2.3 Soil Incubations

SOC decomposability and isotopic signatures (^13^C, ^14^C) of microbial respired CO_2_ were determined by short‐term soil incubations for each depth layer at field‐moist conditions. For LWF forest sites, however, dried and sieved samples were used for incubation, which were rewetted to 60% of their water holding capacity using a 0.5 wt‐% NaCl solution and leaving them at constant moisture levels for 4 weeks to readjust microbial activity. Given that the effect of air‐drying and rewetting on ∆^14^CO_2_ values in soil incubations has been shown to be minimal (Beem‐Miller et al. 2021), we are confident that ∆^14^CO_2_ values are comparable across all soil samples. All soil samples were incubated at 22°C. Depending on the depth layer and their SOC contents, soils were incubated corresponding to dry soil weights between 30 and 250 g. For the incubation, root‐free soil samples were placed in 2 L glass bottles which were then flushed with CO_2_‐free air until all ambient air was removed. Soils were incubated until CO_2_ concentrations were high enough for subsequent ^14^C analysis (~2000 ppm). Depending on the respiration rates, incubation times varied between 1 day and 4 weeks. SOC decomposability was calculated for each soil incubation by integrating respiration rates over the entire incubation period and normalizing them to SOC content. Gas sampling for ^14^C analysis was conducted using a flow‐through system connected to a LI‐COR gas analyser. Before sampling, CO_2_ was scrubbed from all gas tubes and H_2_O traps using soda lime. Air from inside the bottles was pumped through a 2 L air bag (Cali‐5‐Bond, Calibrated Instruments LLC, USA), which was pre‐filled with CO_2_‐free air to maintain the pressure inside the flow‐through system. Sampling was completed once the air from the bottle and the air bag was thoroughly mixed, as indicated by a stable CO_2_ concentration. For ^13^C analysis, additional gas was sampled from each bottle into pre‐evacuated 12 mL Excetainer vials using a 60 mL syringe.

### 2.4 Isotopic Analysis of Gas Samples

The ^13^CO_2_ content of all gas samples was measured using an isotope‐ratio mass spectrometer (IRMS Gas‐Bench II coupled with a Delta‐V Advanced IRMS, Thermo GmbH, Germany). For ^14^C analysis, gas samples were graphitized using an Air Loading Facility (ALF; Gautschi 2017) coupled to an Automated Graphitization Equipment (AGE3, ETH Zurich, Switzerland; Wacker, Němec, and Bourquin 2010) with an integrated zeolite trap to adsorb CO_2_ from the sampling bag. The ^14^CO_2_ content of all gas samples was measured using a MIni radioCArbon DAting System (MICADAS, ETH Zurich, Switzerland; Synal et al. 2007) or a Low Energy AMS (LEA, ETH Zurich & IonPlus AG, Switzerland; Ramsperger et al. 2024). Typical measurement uncertainties were < 2‰. For data evaluation, the standard Oxalic Acid II (OxII) (Mann 1983) and blank material from the ^14^C‐free phthalic anhydride (PhA) were measured alongside the samples and evaluated with the BATS software (Wacker, Christl, and Synal 2010). All ^14^C data in this study are reported in the ∆^14^C notation (Schuur et al. 2016).

Comparisons of stable isotopic and radiocarbon signatures between microbially respired CO_2_ and SOC indicated potential contributions of carbonate weathering to CO_2_ production at certain sites and depths. To minimize biases, we excluded Δ^14^CO_2_ values and ^14^CO_2_‐derived model results from further analysis for sites and depths where estimated carbonate contributions to respired CO_2_ exceeded 5% (*n* = 11 out of a total of 107 site‐depth combinations). For sites where potential carbonate contributions were < 5%, we used carbonate‐corrected Δ^14^CO_2_ values (Appendix S1, Table S2).

### 2.5 Isotopic Analysis of Bulk Soil

Milled soil samples were analysed for total and organic C, total N, as well as δ^13^C and δ^15^N by dry combustion with an automated elemental analyser—continuous flow isotope ratio mass spectrometer system (Euro‐EA 3000, HEKAtech GmbH, Germany, interfaced with a Delta‐V Advanced IRMS, Thermo GmbH, Germany). Samples with δ^13^C values exceeding −25‰, indicative of potential inorganic C contributions, were additionally analysed after fumigation with 37% HCl to remove inorganic C (Walthert et al. 2010).

For ^14^C analysis, potential inorganic C was removed from all samples by fumigation with 37% HCl (Komada et al. 2008). Samples were acidified for 72 h at 60°C and neutralized with NaOH pellets (72 h, 60°C). ^14^C measurements of SOC in bulk soil were performed on a MICADAS (ETH Zurich, Switzerland; Synal et al. 2007) featuring a gas ion source and coupled to an Elemental Analyser (EA vario MICRO cube, Elementar, Germany; Ruff et al. 2010) at the Laboratory of Ion Beam Physics, ETH Zurich, Switzerland. Measurement uncertainties were 6‰–8‰. All glassware was combusted at 550°C for 5 h prior to use.

### 2.6 Soil Mineralogy

Bulk soil samples were analysed for their content of metal phases relevant for mineral SOC stabilization by sequential extraction of Al, Fe, and Mn oxyhydroxides following the approach described in Wasner et al. (2024). In short, 0.5 g of milled bulk soil was shaken horizontally at room temperature for 16 h with a sodium pyrophosphate solution (0.1 M Na_4_P_2_O_7_ × 10 H_2_O and 0.5 M Na_2_SO_4_, pH 10) at a soil: solution ratio of 1:40 to remove organo‐metallic complexes. After extraction, the vials were centrifuged (Sigma 3–16 KL, 10 min, 1700 × g), the supernatant was decanted and filtered through Whatman 41 filter paper. The remaining soil residue was then subjected to a second extraction by shaking it at room temperature for 2 h with a 0.2 M ammonium oxalate solution (0.2 M (NH_4_)_2_C_2_O_4_ × H_2_O and 0.2 M C_2_H_2_O_4_ × 2 H_2_O, mixed at a 1.31:1 ratio) at pH 3 in the dark to prevent photodegradation. The extract was subsequently filtered, diluted to 50 mL with nanopure water, and stored at 4°C until analysis. In oxalate extracts, Al, Fe, and Mn concentrations were measured using ICP‐OES (5100 ICP‐OES, Agilent Technologies, USA). The elemental contents of oxalate extractable Al, Fe, and Mn in bulk soil were summed up and normalized to the SOC content. We refer to the sum of oxalate‐extractable metals (Al, Fe, Mn) as pedogenic oxides and use this measure as an indicator of mineral reactivity.

### 2.7 Analysis of Residual Oxidisable Carbon (ROC)

ROC was measured from bulk soil using a soliTOC EA (Elementar Analysensysteme GmbH, Germany). Depending on the SOC concentration, 10–100 mg of ground soil sample was weighed into a ceramic crucible. Samples were subjected to a ramped temperature program from room temperature to 900°C with three temperature plateaus at 400°C, 600°C, and 900°C following the DIN19539 standard. ROC content is defined as the fraction of CO_2_ produced at temperatures between 400°C and 600°C (Rennert and Herrmann 2022). The evolved CO_2_ was quantified by non‐dispersive infrared detection. A low and a high organic C standard as well as pure CaCO_3_ (all provided by Säntis Analytics) were run at regular intervals to monitor the instrument's accuracy, which was determined to be 98.9% ± 0.6%.

### 2.8 Modelling of Transit Time and System Age Using Radiocarbon Signatures in Respired CO_2_ and Bulk Soil

We applied a compartmental modelling approach to determine transit times and system ages of SOC. All models were implemented using the SoilR package (version 1.2.107, Sierra et al. 2012, 2014) and constrained with observed values of ^14^C in respired CO_2_ and bulk soil (Beem‐Miller 2023). We assumed steady‐state conditions across all land‐use types to enable consistent estimation and comparison of transit times and system ages. In steady‐state conditions, the input flux can be set to an arbitrary value of 1 (Schuur et al. 2016).

SOC dynamics in a compartmental model can be approximated as a system of linear differential equations (e.g., Sierra et al. 2012; Sierra and Müller 2015):

(1) $$\frac{dC\left(t\right)}{dt}=I+A\times C\left(t\right)$$

where ***C**(t)* is a vector representing the C stocks in *n* pools at time *t*, ***I*** is a vector of constant C inputs to each pool, and **A** representing an *n* × *n* time‐invariant decomposition matrix which contains the inverse of the decomposition rate *k* for each pool *n* in its main diagonal, and coefficients representing the rate of C transfer from one pool to another in the off‐diagonals (Sierra et al. 2012, 2014; Sierra and Müller 2015). SO^14^C dynamics can subsequently be described as:

(2) d14C(t)dt=I14C(t)+A×C14(t)−λC14(t)

where ***I***_*14C(t)*_ is the time‐varying ^14^C of C inputs and *λ* is the radiocarbon decay constant (1/8267 [year^−1^]).

Differences in the Δ^14^C signatures of microbially respired CO_2_ and of SOC in bulk soil of the same sample provide evidence that the soil matrix is not a homogeneous pool. We thus implemented a two‐pool model (one slow and one fast pool) and applied a series model structure where all C inputs enter only to the fast pool, and then are either decomposed or transferred to the slow pool at a rate defined by the transfer coefficient α (Manzoni et al. 2009; Sierra et al. 2014). The series model structure for a two‐pool model can be expressed as:

(3) $$\frac{dC}{dt}=I\left(\begin{array}{c} 1\\ 0 \end{array}\right)+\left(\begin{array}{cc} -k_{f} & 0\\ \alpha_{s,f}k_{f} & -k_{s} \end{array}\right)\times \left(\begin{array}{c} C_{f}\\ C_{s} \end{array}\right)$$

where *–k*_*f*_ and *–k*_*s*_ are the decomposition rate constants of the fast and slow pool, respectively, and *α*_s,f_ represents the fraction of C transferred from the fast to the slow pool upon turnover. ∆^14^C values of C inputs were derived from existing bomb curve data sets of atmospheric CO_2_ (Hua et al. 2022). Bomb curve data sets used in the modelling were adjusted to each site and depth increment to account for site‐specific atmospheric ^14^CO_2_ levels, as well as time lags induced by C inputs (Appendix S2, Table S3).

We applied a three‐step process to determine the optimal model parameter set for each site and depth combination using observed ^14^C values for respired CO_2_ and bulk SOC as constraints (Beem‐Miller 2023). We first fit an initial parameter set (*k*₁ for the fast pool, *k*_2_ for the slow pool, and *α*_2,1_) manually to obtain reasonable starting values. These initial estimates were refined through nonlinear least‐squares optimization using the function *modFit* from the FME package (Version 1.3.6.3; Soetaert and Petzoldt 2010). Finally, the optimized parameter estimates from *modFit* were used to initialize Markov Chain Monte Carlo (MCMC) simulation with 10,000 iterations. We ran an adaptive MCMC simulation using the function *modMCMC* (FME package), with delayed rejection turned on to improve parameter convergence. The MCMC approach is particularly useful for quantifying uncertainty associated with parameter equifinality, that is, parameter combinations that are equally likely given the data constraints (Beem‐Miller 2023; Sierra et al. 2015; Stoner et al. 2021). Model performance in this study was restricted by the limited temporal resolution of the ^14^C data, that is, only a single time point was available for constraining the model. We quantified the degree of parameter equifinality by assessing correlations among the MCMC posterior parameter distributions at a subset of sites and depths as well as with the FME function *collin* (Sierra et al. 2015). More detailed information on the probability distribution functions for transit times and system ages are presented in Appendix S3.

We quantified the uncertainty of transit times and system ages by calculating mean and median values from the probability distribution functions, as well as the standard deviations of these values, using a random subset of 200 parameter sets drawn from the posterior distribution of the MCMC simulation. For all models, the median transit times and median system ages were consistently lower than their respective mean values because the mean is more sensitive to the skewness of the age distribution. We thus used median values for subsequent data analysis as they are likely a more realistic measure of these distributions (Sierra et al. 2018).

### 2.9 Calculation of a SOC Vulnerability Index

To assess the relationship between SOC persistence (represented by system age) and SOC stability (represented by %‐ROC in SOC) across land‐use types and soil depths, we calculated a SOC vulnerability index (VI) as:

(4) $$\mathrm{VI}=\frac{\ln\left(1+\text{system}\;\mathrm{age}\right)}{\%-\mathrm{ROC}\;\text{in}\;\mathrm{SOC}}$$

A low system age‐to‐%‐ROC ratio (i.e., ~0.1) indicates that SOC is both persistent and stable, whereas a high ratio (i.e., > 1.0) suggests SOC vulnerability, meaning SOC is persistent but lacks stabilization. We applied a natural log‐transformation—ln(1 + system age) – to system age to account for the disproportionate influence of extremely old SOC, particularly in managed peatlands and subsoils, while expanding the scale for younger SOC. Adding 1 ensured that system ages of 1 (i.e., in the litter layer of some sites) did not result in a log‐value of zero.

### 2.10 Statistical Analysis

To investigate how Δ^14^C values of microbially respired CO_2_ and SOC in bulk soil, their corresponding transit times and system ages, as well as MAT and soil properties varied across land use and soil depth, we fitted linear mixed‐effects (LME) models and included site as a random effect (R package lmerTest; Kuznetsova et al. 2017). Response variables were transformed in case the assumption of normal distribution (Shapiro–Wilk normality test) or homoscedasticity (Levene's test and plots of fitted vs. residuals) was violated. We conducted an analysis of variance (ANOVA) on the fixed effects (land‐use type and soil depth) of the models to assess their significance and interaction. Following the main effects, post hoc pairwise comparisons were conducted using estimated marginal means (EMMs) (R package emmeans, version 1.10.4; Lenth 2024) to assess significant differences between land‐use types within each depth and vice versa, adjusted for multiple comparisons using Tukey's method. To examine variations in transit times and system ages within each land‐use type, we fitted a LME model with depth as a random effect. An ANOVA was then performed on the fixed effects (age type [transit time or system age] and land‐use type), followed by post hoc pairwise comparisons to identify land‐use types where transit times and system ages differed significantly. We further fitted linear regression models (LM) to assess the significance and effect size of the link between soil properties and transit time and system age for each land‐use type. For grassland sites (including temperate and alpine), we additionally fitted LMs to investigate the effect of MAT on transit times and system ages. We excluded MAP from the statistical analysis, as data was available only from one station for all alpine grasslands and one station for all managed peatlands.

Principal component analysis (PCA) was performed on scaled and centred variables (R package psych, version 2.4.12; Revelle 2007) to explore how climate, SOC characteristics, and soil physicochemical properties characterize the different land‐use types. It allowed clustering the land‐use types based on the measured variables. Additionally, a correlation matrix was generated to identify relationships among these properties.

## 3 Results

### 3.1 Climate and Soil Properties Across Land‐Use Types and Soil Depth

**FIGURE 2:**
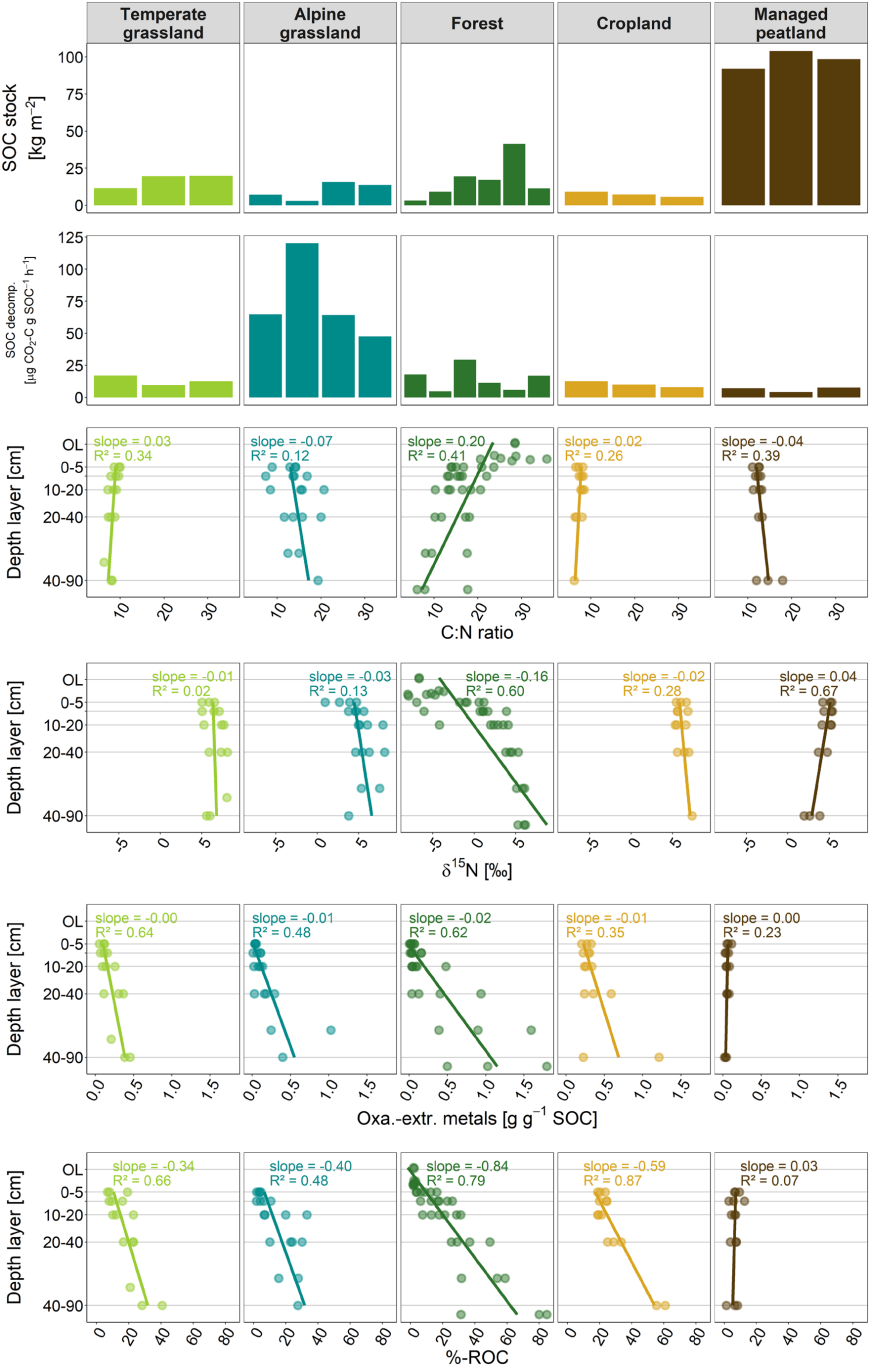
SOC characteristics and soil physicochemical properties of land‐use types, including (from uppermost to lowermost panels) total SOC stocks (kg m^−2^) and total SOC decomposability (μg CO_2_–C g SOC^−1^ h^−1^) for investigated sites within each land‐use type, as well as C:N ratios, δ^15^N values (‰), oxalate‐extractable metals (g g^−1^ SOC), and ROC in SOC (%) for which linear relationships with soil depth are shown.

Climate, SOC characteristics, and soil physicochemical properties differed significantly across land‐use types (*p*_oxa.‐extr. metals_ = 0.040, *p*_all other variables_ < 0.0001) and soil depths (*p*_all variables_ < 0.0001; Table S7). However, it should be noted that the differences between land‐use types were primarily driven by managed peatlands and alpine grasslands (Table S7), while temperate grasslands, forests, and croplands shared more similar soil properties and climate (Figure S2, Tables S8 and S9). Alpine grasslands were primarily distinguished by their low MAT and having the highest SOM decomposability (Figure S2). Forests showed the most pronounced depth‐related gradients in their SOC characteristics, with the highest C:N ratios in the organic layer and the highest contents of pedogenic oxides and %‐ROC in subsoil (Figure 2, Figure S2). Managed peatlands were characterized by high C:N ratios throughout the soil profile and the lowest levels of pedogenic oxides and %‐ROC in SOC across all land‐use types (Figure 2, Table S12).

Alpine grasslands located at the highest elevation experienced the lowest MAT, while managed peatlands had the highest. Temperate grasslands, forests, and croplands showed similar MATs (Table S1, Figure S2). Soil pH was lowest in forests and alpine grasslands, and highest in croplands (Figure S3, Table S1). Clay contents were lowest in alpine grasslands and forests (Figure S3). Total SOC stocks were greatest in managed peatlands (98 kg SOC m^−2^), followed by forests and temperate grasslands, both with average SOC stocks of 16.9 kg SOC m^−2^. Stocks were lowest in alpine grasslands (9.9 kg SOC m^−2^) and croplands (7.3 kg SOC m^−2^) (Figure 2, Table S12). SOC decomposability was highest in alpine grasslands (72 μg CO_2_–C g SOC^−1^ h^−1^), and lowest in croplands (10 μg CO_2_–C g SOC^−1^ h^−1^) and managed peatlands (6 μg CO_2_‐C g SOC^−1^ h^−1^; Figure 2, Table S12). %‐ROC in SOC increased markedly with soil depth across all land‐use types, except in managed peatlands (*p* < 0.001; Figure 2, Table S9). The highest contributions of ROC to SOC were found in the subsoils of forests and croplands with values up to 80%, whereas managed peatlands contained minor amounts (< 10%; Figure 2, Table S12). Pedogenic oxide contents exhibited a similar pattern as %‐ROC.

### 3.2 Radiocarbon, Transit Times and System Ages Across Land‐Use Types and Soil Depths

**FIGURE 3:**
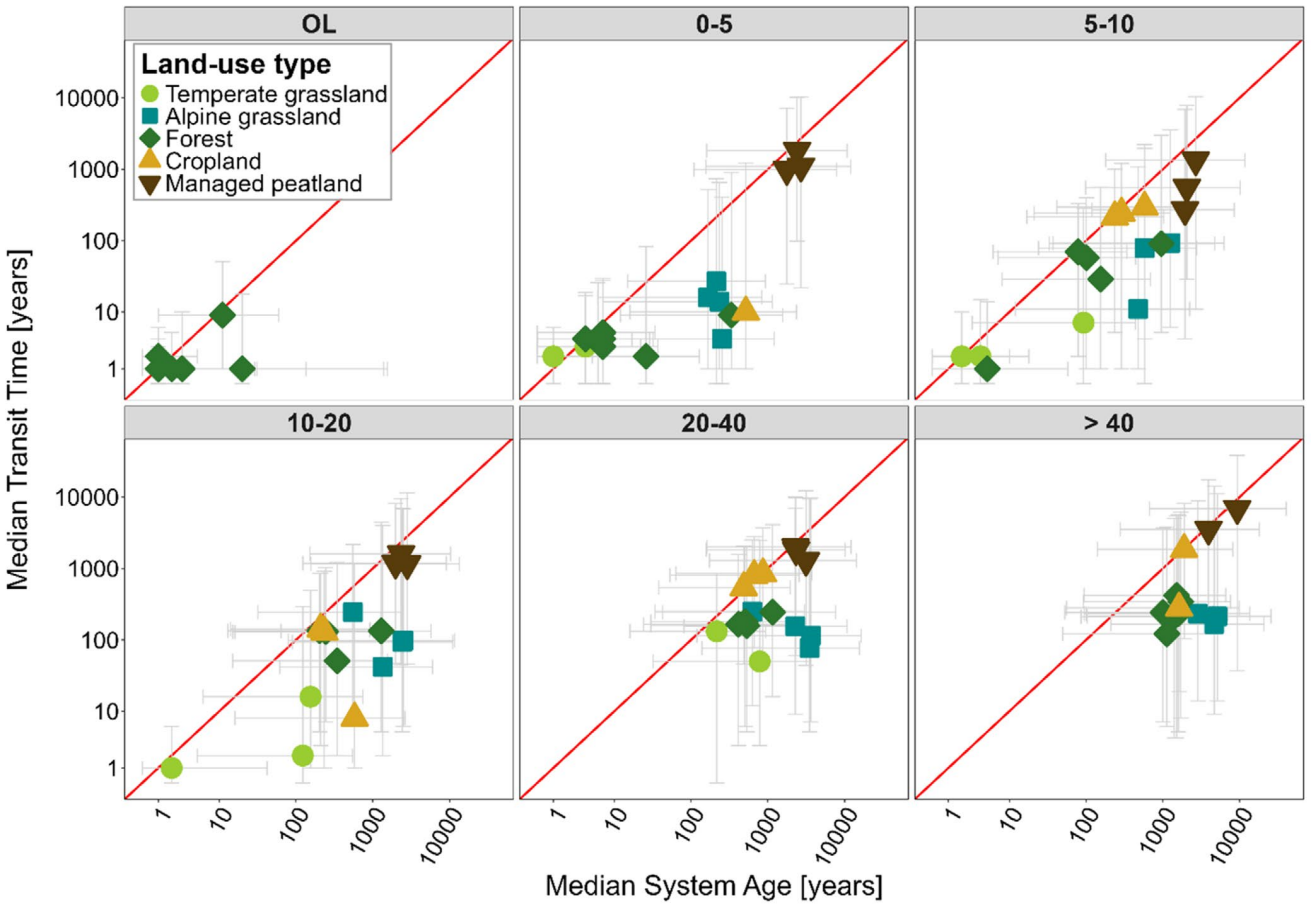
Median transit times versus median system ages (years) across soil depths (cm) and land‐use types, with the 25th and 75th percentiles shown as a measure of variability. The red line indicates the 1:1 relationship. OL = organic layer.

Averaged across all land‐use types and soil depths, respired CO_2_ was younger (median transit time: 540 years) than bulk soil SOC (median system age: 1580 years; Figure 3, Table 1). The differences between transit times and system ages were greatest in alpine grasslands and forests (*p* < 0.0001) and least in croplands and managed peatlands (*p*_cropland_ = 0.014, *p*_managed peatland_ = 0.021; Figure 3, Table S6).

**TABLE 1:** Radiocarbon data and modelled ages across land‐use types and soil depths. Profile values for transit times and system ages were calculated by weighting each depth layer based on its contribution to the total SOC decomposability and SOC stock, respectively. Soil depth > 40 cm comprises a maximum depth up to 90 cm. Values are reported as means ± standard deviation.

Land‐use type	Depth (cm)	Transit times		System ages	
		Δ^14^CO_2_ (‰)	Median (years)	Δ^14^C‐SOC (‰)	Median (years)
Temperate grassland	Total profile	−4 ± 20	11 ± 8^a^	−40 ± 98	732 ± 653
	0–5	−1 ± 12	3 ± 1	2 ± 22	4 ± 3
	5–10	4 ± 15	4 ± 3	13 ± 24	33 ± 52
	10–20	−11 ± 34	6 ± 8	20 ± 9	93 ± 81
	20–40	−9 ± 26	91 ± 58	−55 ± 52	484 ± 285
	> 40	—	—	−182 ± 144	1517 ± 1286
Alpine grassland	Total profile	−20 ± 51	73 ± 44	−181 ± 161	1678 ± 1061
	0–5	35 ± 21	15 ± 9	−3 ± 12	217 ± 37
	5–10	6 ± 41	61 ± 44	−75 ± 68	649 ± 419
	10–20	−17 ± 19	120 ± 87	−207 ± 106	1708 ± 933
	20–40	−46 ± 16	149 ± 74	−274 ± 138	2513 ± 1395
	> 40	−99 ± 27	205 ± 34	−401 ± 72	4218 ± 1193
Forest	Total profile	10 ± 42	47 ± 49	−23 ± 99	256 ± 226
	Organic layer	10 ± 29	2 ± 3	21 ± 43	5 ± 7
	0–5	48 ± 21	5 ± 2	56 ± 49	65 ± 133
	5–10	43 ± 20	50 ± 35	22 ± 76	259 ± 393
	10–20	20 ± 34	91 ± 60	−10 ± 94	421 ± 500
	20–40	−6 ± 24	188 ± 39	−86 ± 57	652 ± 337
	> 40	−52 ± 25	262 ± 105	−183 ± 33	1394 ± 274
Cropland	Total profile	−81 ± 62	531 ± 338	−77 ± 74	728 ± 441
	0–5	−30	10	−41 ± 29	356 ± 141
	5–10	−46 ± 24	253 ± 41	−42 ± 36	369 ± 184
	10–20	−48 ± 14	94 ± 74	−36 ± 42	342 ± 208
	20–40	−119 ± 15	731 ± 165	−84 ± 36	673 ± 190
	> 40	−153 ± 127	1069 ± 1144	−232 ± 18	1763 ± 190
Managed peatland	Total profile	−223 ± 97	2048 ± 688	−307 ± 95	4501 ± 2249
	0–5	−191 ± 35	1308 ± 460	−263 ± 42	2293 ± 477
	5–10	−144 ± 55	725 ± 556	−268 ± 38	2227 ± 398
	10–20	−197 ± 20	1313 ± 249	−271 ± 38	2377 ± 466
	20–40	−232 ± 14	1702 ± 365	−289 ± 37	2591 ± 480
	> 40	−417 ± 120	5203 ± 2373	−442 ± 179	5761 ± 3077

^a^ Profile value for transit times in temperate grasslands likely underestimated, as soil depth > 40 cm could not be considered due to missing values (removed due to potential carbonate contribution).

Overall, Δ^14^C values of microbially respired CO_2_ and SOC in bulk soil, as well as their corresponding median transit times and system ages differed significantly across land‐use types and soil depths (*p* < 0.0001, Table S7). Averaged across the profile, transit times increased from 11 ± 8 years in temperate grasslands through forests, alpine grasslands, croplands, to 2048 ± 688 years in managed peatlands (Table 1). System ages increased from 256 ± 226 years in forests through temperate grasslands, croplands, alpine grasslands, to 4501 ± 2249 years in managed peatlands (Table 1).

**FIGURE 4:**
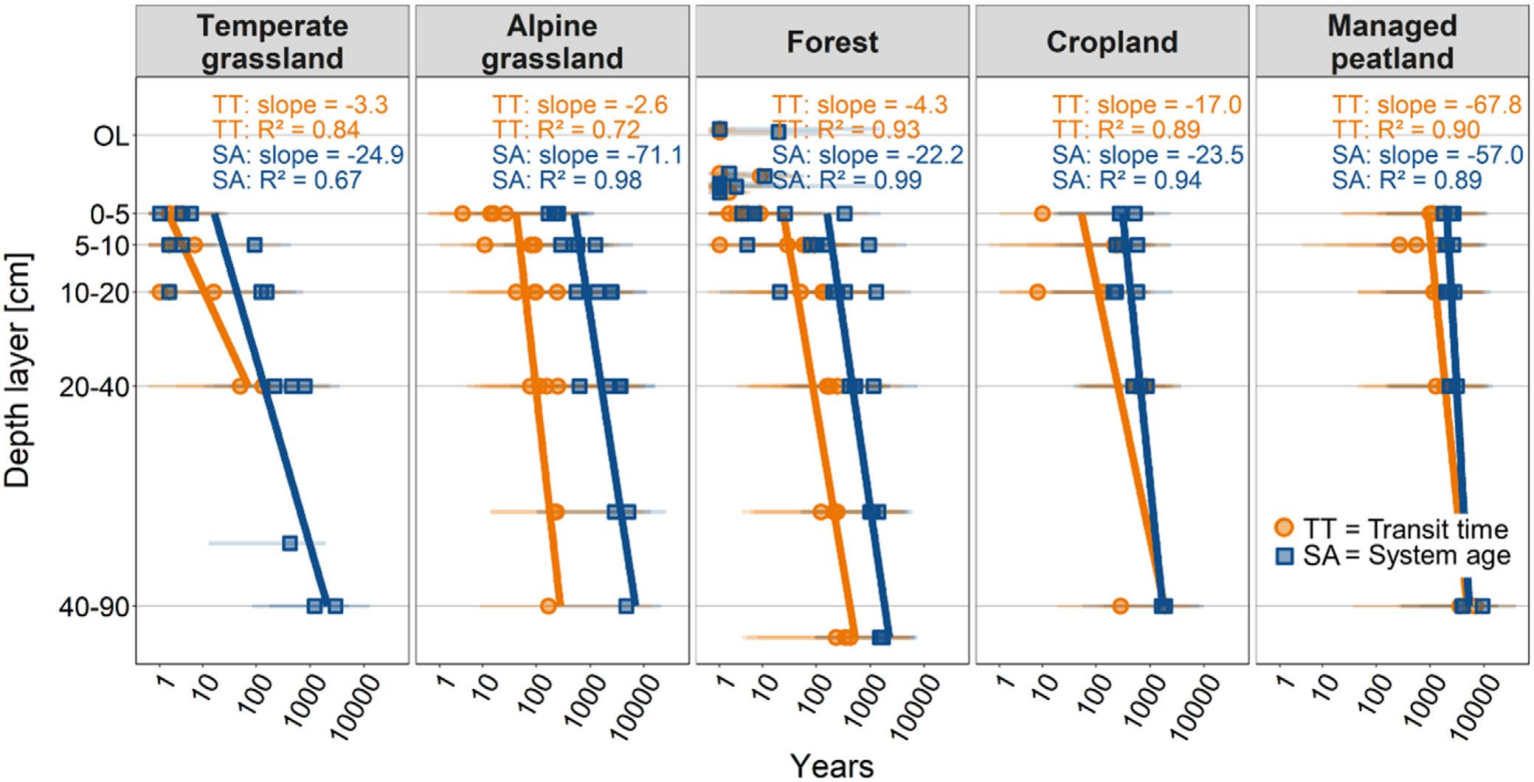
Modelled median transit times (TT) and system ages (SA) across soil depth for each land‐use type (logarithmic scale), with the 25th and 75th percentiles shown as a measure of variability. Linear regressions for transit times and system ages with mineral soil depth (excluding organic layers in forests) were significant. Corresponding *p*‐values are provided in Table S10. OL = organic layer. Corresponding Δ^14^C values of microbially respired CO_2_ and bulk SOC are presented in Figure S4.

The influence of land use on transit times and system ages was more pronounced in upper soil layers (< 20 cm soil depth) and lost its significance for system ages in deeper soil layers (> 20 cm; Table S8). Generally, transit times and system ages significantly increased with soil depth for all land‐use types (*p* ≤ 0.083, Table S10). This increase was more pronounced for system ages (mean rate = 40 ± 23 years cm^−1^ soil depth) than for transit times (mean rate = 19 ± 28 years cm^−1^ soil depth; Figure 4). Forests exhibited the least pronounced depth‐related increase of system age (~22 years cm^−1^ soil depth) while alpine grasslands showed the largest increase (~71 years cm^−1^ soil depth). Transit times in temperate and alpine grasslands and in forests increased at a similarly low rate with soil depth (~3–4 years cm^−1^ soil depth) and the increase was highest in managed peatlands (~68 years cm^−1^ soil depth; Figure 4).

### 3.3 Relationships Between Transit Time and System Age With Soil Properties and MAT Across Land‐Use Types

**FIGURE 5:**
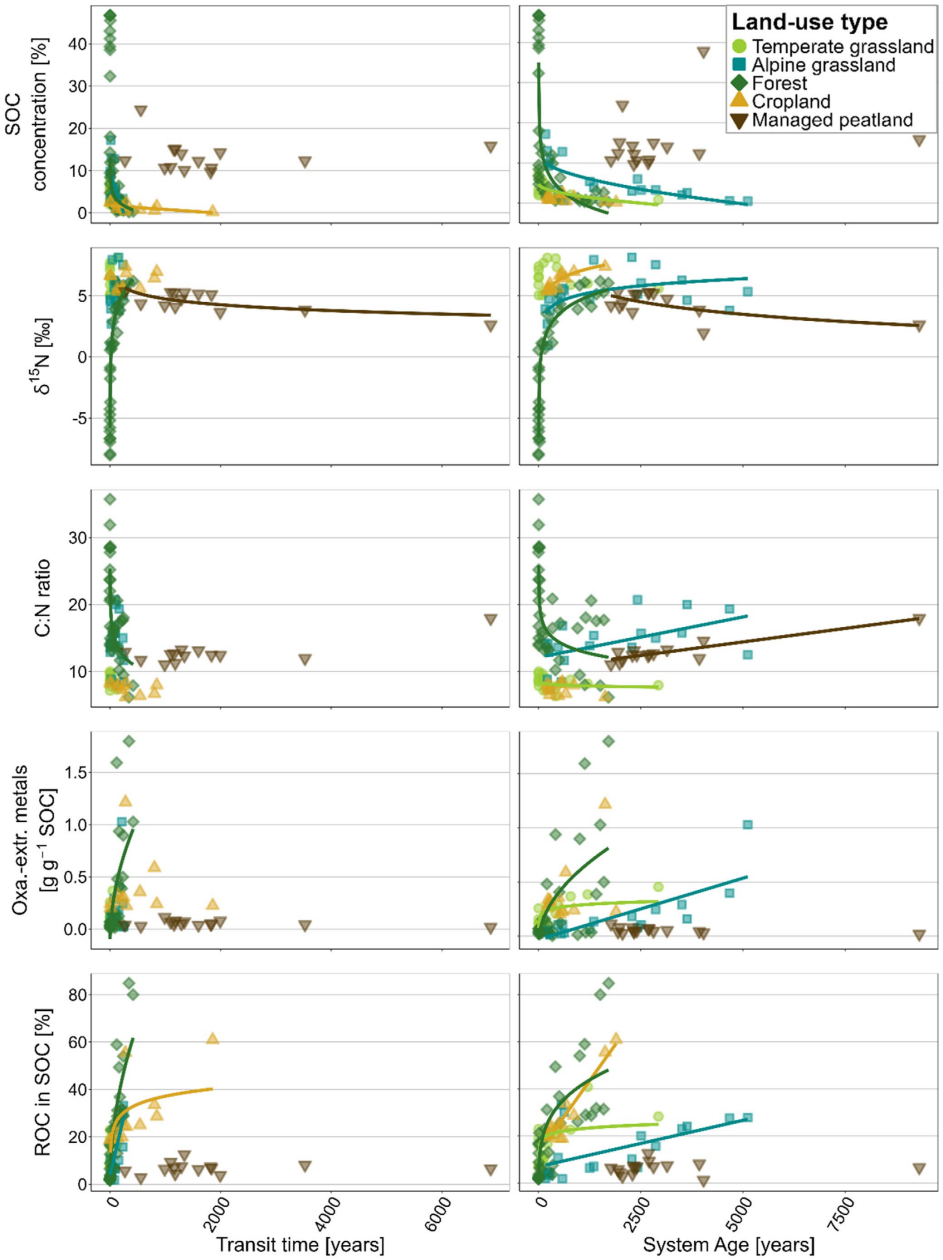
Relationships of soil properties with transit times and system ages across land‐use types. Fits were plotted to match transformations applied in statistical analysis. Only significant relationships are shown (*p* < 0.05). Corresponding *p*‐values, *R* ^2^, and coefficients are provided in Table S10.

Generally, measured variables—including SOC concentrations, C:N ratios, δ^15^N values, %‐ROC in SOC, and pedogenic oxides—were more strongly related to system ages than to transit times (Figure 5, Table S10). While SOC concentrations generally decreased with increasing system age, pedogenic oxide contents and %‐ROC showed consistently positive correlations with system age except in managed peatlands (Figure 5, Table S10). In contrast, the relationships between C:N ratios and δ^15^N values and system age varied across land‐use types (Figure 5, Table S10). While C:N ratios increased and δ^15^N values decreased with system age in temperate grasslands and forests, they showed the opposite relation in managed peatlands. In croplands, both transit times and system ages showed little or no relationships with soil physicochemical properties (Figure 5, Table S10). Clay content and pH showed little predictive power for transit times and system ages across all land‐use types (Table S10).

For the studied grassland sites (including temperate and alpine grasslands), spanning a MAT gradient from −1.2°C to 9.9°C, system ages showed a stronger and more significant correlation with MAT than transit times. However, this relationship became less significant with increasing soil depth (Table S11, Figure S5).

### 3.4 Vulnerability Across Land‐Use Types and Soil Depths

**FIGURE 6:**
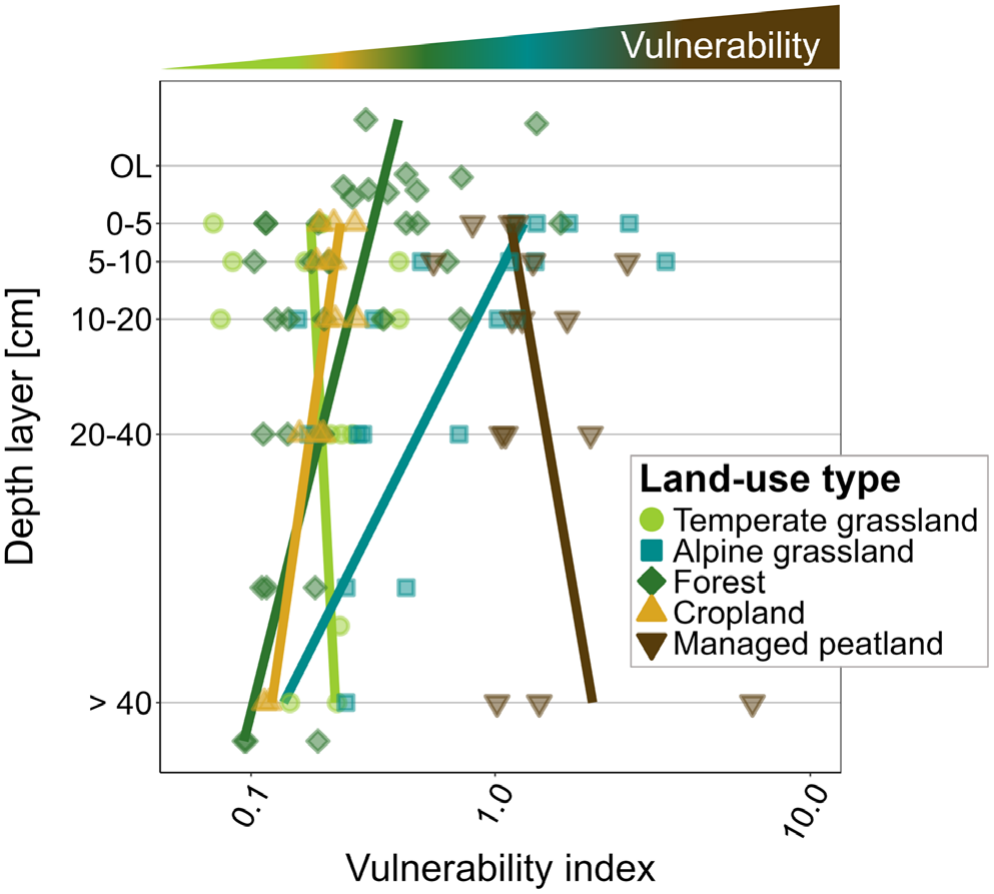
SOC vulnerability index (VI) across land‐use types and soil depths. Lines along depth profiles serve as visual guides to indicate trends in vulnerability. Note that the *x*‐axis is log‐transformed. OL = organic layer.

Overall, the vulnerability index of SOC (VI) – relating the ln of system age to %‐ROC (Equation (4))—showed distinct differences between land‐use types (*p* = 0.001, Table S7). Managed peatlands exhibited the highest VI throughout the soil profile (Figure 6). In alpine grasslands, the VI was high in topsoil layers but significantly decreased with soil depth (*p* < 0.0001; Figure 6, Table S9). Forests showed a high vulnerability in the organic layer, followed by a significant decrease with soil depth (*p* = 0.013; Table S9) to similarly low indices as in temperate grasslands and croplands.

## 4 Discussion

### 4.1 ^14^C‐Based Transit Times and System Ages Across Land‐Use Types and Soil Depths

Our study revealed significant differences in ^14^C‐derived ages in soil‐respired CO_2_ (transit times) and bulk SOC (system ages) among five dominant land‐use types across Switzerland, ranging from 2 years in the organic layer of forest soils to 5760 years in subsoils of managed peatlands (Table 1). Linking these ^14^C‐derived age metrics to soil properties and climate variables showed that SOC persistence is associated with different mechanisms depending on land use—either through the inhibition of decomposer activity under unfavourable environmental conditions or through limited SOC accessibility due to organo‐mineral stabilization.

Across all land‐use types and soil depths, microbially respired CO_2_ was younger (average transit time: 540 years) than bulk SOC (average system age: 1580 years). This indicates that the respired CO_2_ primarily originates from the younger, more labile fraction of SOC, readily available for microbial decomposition (Dungait et al. 2012; González‐Domínguez et al. 2019). Consequently, transit times reflect actively cycling SOC. In contrast, bulk SOC encompasses a broader array of compounds with a wide range of ages, including older and more persistent SOC fractions. Thus, system age can serve as an indicator of SOC persistence (Sierra et al. 2018).

Alpine grasslands showed the greatest discrepancy between transit times and system ages (Figure 3). We attribute this to the harsh climate in these ecosystems > 2300 m a.s.l., with a very short growing season and prolonged freezing temperatures that inhibit microbial decomposition (Table S4; D'Alò et al. 2021; Margesin et al. 2009). Reduced microbial activity may lead to the preferential decomposition of the most labile, ‘young’ SOC fraction, while the remaining C accumulates and persists, potentially becoming stabilized through mineral associations in deeper soil layers and thereby leading to older system ages (Figure 4). In contrast to alpine grasslands, croplands and managed peatlands exhibited greater SOC homogeneity as indicated by similar transit times and system ages in both systems, both of which being rather old (Figure 3). In the case of croplands, this is primarily due to soil tillage and low C inputs, which have resulted in a loss of young, labile SOC (Keel et al. 2019), and the development of relatively homogeneous soils. In the case of managed peatlands, SOC homogeneity is predominantly related to the disappearance of the upper most peat layers during centennial decomposition induced by drainage, leaving behind mostly old C for microbial decomposition (Leifeld et al. 2011). Taken together, we found that transit times and system ages as well as their relationship to each other can provide valuable insights into the differences in SOC dynamics across land‐use types.

### 4.2 Drivers of Active SOC Cycling and SOC Persistence Across Land‐Use Types and Soil Depths

Soil depth was the most consistent predictor of transit times and system ages (Table S10). The significant increase in the age of respired CO_2_ and especially bulk SOC with depth in all land‐use types (Figure 4) aligns with findings from previous studies (e.g., Balesdent et al. 2018; Heckman et al. 2022; Shi et al. 2020). Increasing mineral reactivity (i.e., pedogenic oxides) and thermal stability (i.e., %‐ROC in SOC) combined with decreasing C:N ratios and increasing δ^15^N values which reflect progressing microbial SOM transformation with soil depth (Figure 2), show that SOC ageing with depth results from the combined influence of various depth‐related processes (Heckman et al. 2022), including vertical transport (Kaiser and Kalbitz 2012), enrichment in microbial metabolites in SOC, microbial energy and substrate limitations, as well as increasing sorption capacity for organo‐mineral associations (Ahrens et al. 2020; Button et al. 2022; Hicks Pries et al. 2023). The less pronounced depth gradient of transit times compared to system ages (Figure 4) suggests that subsoils receive inputs of more recent C (e.g., from deeper roots; Sierra et al. 2024), which is rapidly mineralized (Xiao et al. 2022). In contrast, the stronger increase in system age with depth indicates greater SOC persistence.

Our study documents a dominant control of reactive mineral surfaces on SOC persistence in mineral soils (Figure 5), which also explains why the influence of land use on system age was less pronounced in subsoils compared to topsoils (Table S8). Given the strong correlation between pedogenic oxides and %‐ROC in SOC (Figure S2; *p* < 0.0001, *R* ^2^ = 0.56), we infer that ROC in the studied soils primarily represents a highly persistent mineral‐associated SOC fraction (Grant et al. 2019; Hemingway et al. 2019), rather than petrogenic compounds (Mittelbach et al. 2025). While MAOM fractions may span a broad range of thermal stabilities (Giannetta et al. 2018; Stoner, Trumbore, et al. 2023; Stoner, Schrumpf, et al. 2023), ROC in our analysis is restricted to 400°C–600°C and thus preferentially captures the more thermally stable fraction of MAOM. Among the considered soil physicochemical properties, %‐ROC in SOC was the most consistent predictor of transit time and system age for all land‐use types, except managed peatlands (Figure 5). The stronger predictive power of %‐ROC in SOC as compared to pedogenic oxides may reflect the influence of Ca^2+^, which plays a key role in SOC stabilization in calcareous soils beyond pedogenic oxides (Rowley et al. 2018). Additionally, refractory components such as pyrogenic C might contribute to high %‐ROC, long transit times, and old system ages. However, a survey of 54 Swiss forest soil profiles found that pyrogenic C contributed, on average, less than 5% to total topsoil SOC (González‐Domínguez et al. 2019). Although forest soils showed the highest mineral reactivity and thermal stability in subsoil layers, system age increased the least with soil depth in forests. One likely reason could be the greater C inputs to subsoil by deeper roots in forests compared to other land‐use types (Jackson et al. 1996). In soils of managed peatlands, mineral reactivity and thermal stability were lowest across all land‐use types despite longest transit times and oldest system ages, suggesting that mineral protection of SOM was of minor importance in the context of their high SOC persistence. In our data set, clay content and soil pH played a minor role to explain SOC persistence, which is consistent with previous studies (Chen et al. 2021; Rasmussen et al. 2018; Wasner et al. 2024).

Since temperate grasslands, forests, croplands, and managed peatlands were all located at comparable climatic conditions (Table S1), it was only possible to evaluate temperature effects on transit time and system age for grasslands, which span a gradient in MAT of −1.2°C to 9.9°C from alpine to temperate sites. Transit times and system ages increased significantly with decreasing MAT (Table S11, Figure S5), likely related to reduced microbial activity in colder soils (Liu et al. 2018). Transit time was less sensitive to MAT than system age, consistent with observations at the global scale (Xiao et al. 2022). This indicates that temperature has a stronger effect on the long‐term transformation to persistent SOC than on actively cycling SOC. The effect of MAT on SOC persistence decreased with soil depth (Table S11), likely due to increasing mineral protection of SOC, which attenuates the effect of temperature (Gentsch et al. 2018), along with enhanced microbial energy and substrate limitations at greater soil depths (Ahrens et al. 2020).

Overall, our findings suggest that both soil mineralogy and MAT are key determinants of SOC persistence across land‐use types except in managed peatlands. Mineral associations contribute to increasing system ages with depth, while colder temperatures enhance SOC persistence by limiting microbial activity.

### 4.3 Vulnerability and Destabilization of SOC Across Land‐Use Types

SOC persistence is governed by multiple processes, including the suppression of decomposer activity under adverse environmental conditions, the preservation of recalcitrant organic compounds, and the reduced accessibility of SOC due to stabilization via organo‐mineral interactions (Cotrufo and Lavallee 2022; Schmidt et al. 2011). In this study, we made use of the large span in soil properties and ^14^C‐derived ages to identify dominant mechanisms driving SOC persistence across contrasting land‐use types. By integrating transit times and system ages with indicators of biological and thermal stability, we developed a conceptual framework to assess SOC vulnerability to environmental change across the studied land‐use systems.

System ages revealed that SOC persistence was most pronounced in cold (i.e., alpine grasslands) and oxygen‐limited conditions (i.e., peatlands in their natural condition) (Table 1, Figure 4). At the same time, alpine grassland topsoils and managed peatland soils showed very low thermal stability and low mineral reactivity, which implies that physiological inhibition of microbial decomposition was the most important driver of the high SOC persistence in these soils. Alpine grassland soils were further characterized by low biological stability and large amounts of readily decomposable SOC in topsoils (Table 1), as indicated by high SOC decomposability, higher C:N ratios, and lower δ^15^N values as compared to temperate grasslands (Figure 2). The combination of low biological stability and longer transit times, along with low thermal stability and at the same time old system ages (Figure 6) indicates that alpine topsoils are highly vulnerable to climate warming, which would perturb the conditions that led to the long‐term accumulation of readily decomposable SOC. In subsoils of alpine grasslands, however, lower SOC decomposability together with higher mineral reactivity and higher thermal stability suggests that a greater proportion of SOC is mineral‐associated and stabilized (Figure 2), and thus likely less vulnerable to increasing temperatures (Gentsch et al. 2018; Rocci et al. 2021).

The managed peatlands studied here provide evidence that drainage and subsequent aeration disrupted SOC dynamics, resulting in the loss of largely plant‐derived, easily decomposable SOC. Despite exhibiting the oldest system ages among all land‐use types (Figure 4), managed peatlands displayed the lowest thermal stability throughout the soil profile (Figures 2 and 6). This supports the notion that managed peatlands lack a stable, protected SOC fraction (Paul et al. 2021). In combination with exceptionally high and relatively homogeneous SOC stocks (Figures 2 and 3), this renders peatlands particularly vulnerable to C losses driven by disturbances imposed by land‐use conversion and drainage. At the same time, managed peatlands exhibited high biological stability, reflected by low SOC decomposability, particularly in topsoils. Alongside long transit times, this suggests advanced degradation and losses of the younger, readily available SOC fractions—likely resulting from the ~150 year‐long aeration and tillage. We infer that the remaining SOC pool is chemically more recalcitrant but lacks thermal stability, suggesting that while its decomposition is retarded, it is not inherently protected and remains vulnerable to further degradation. The observed increases in SOC decomposability, C:N ratios, and δ^15^N values with soil depth in managed peatlands indicate that subsoil organic matter is less degraded, rendering these layers particularly susceptible to enhanced decomposition under future drainage.

Although less pronounced than in managed peatlands, our data revealed that the conversion of natural ecosystems to croplands has destabilized younger, labile SOC, resulting in longer transit times and older system ages in the remaining SOC, particularly in topsoils. In support, long‐term experiments document substantial SOC losses from cropland soils that had previously been used as grasslands (Keel et al. 2019; Luo et al. 2020; Poeplau and Don 2013). Following the depletion of labile SOC, the remaining SOC—stabilized through organo‐mineral associations—persists and can be assumed to be comparatively less vulnerable to disturbances.

SOC in forest soils exhibited high vulnerability in the organic layer but not in the mineral soil (Figure 6). Although the vulnerable organic layer typically accounts only for a small proportion of the total SOC pool, it can accumulate substantial C stocks in certain alpine environments, reaching up to ~12 kg C m^−2^ at the Beatenberg site in this study. In a survey of Swiss forest soils, Mayer et al. (2023) found that C stored in organic layers is indeed highly vulnerable as it is rapidly lost following disturbances such as windthrow. In the mineral soils of forests, system age was closely related to mineral reactivity, indicating that interactions with reactive minerals are the key mechanism underlying their low vulnerability.

### 4.4 Future Directions of ^14^C‐Based Assessments of SOC Dynamics

This study provides a comprehensive dataset of ^14^C isotopic signatures and soil properties from soils of five dominant land‐use types across Switzerland. These data reflect typical ecoregion‐specific land‐use patterns developed over centuries and shaped by climate and socio‐economic factors. Our data provide insights into the long‐term impacts of land use on active SOC cycling and SOC persistence. Although, to our knowledge, this ^14^C‐based study is unprecedented with respect to the number of sites and diversity of land‐use types investigated, the spatial and temporal coverage remains limited. Increasing the number of replicates and additional ^14^C time points would improve the accuracy of the modelled transit times and system ages, as single ^14^C time points often result in limited model convergence and strong correlations among constrained model parameters (Sierra et al. 2014). To overcome this challenge, future research should prioritize ^14^C time series. Nevertheless, we believe the findings from this study provide motivation for further investigations adopting the conceptual framework developed here to assess controls on the persistence and vulnerability of SOC. Additionally, we encourage future research to apply the empirical vulnerability index developed in this study in a more universal and mechanistic way by linking SOC age to activation energies of thermal fractions, based on the Arrhenius principle. Recent works which have applied temperature‐resolved thermal analysis to obtain activation energies and ^14^C values of bulk SOC and soil fractions (Hemingway et al. 2019; Stoner, Schrumpf, et al. 2023) could provide a basis to assess SOC stability and vulnerability in such a way. For instance, older C released at lower activation energies could indicate greater SOC vulnerability.

## 5 Conclusions

Radiocarbon measurements of microbially respired CO_2_ and bulk SOC revealed significant differences across land‐use types with notably old ^14^C‐derived ages observed in managed peatlands and alpine grasslands. By linking ^14^C‐based system ages to soil and climate attributes, we were able to elucidate the dominant mechanisms underlying SOC persistence across these land‐use types. In temperate grasslands, forests, and croplands, system ages were most closely associated with thermal stability (i.e., %‐ROC in SOC) and mineral reactivity (i.e., pedogenic oxides) – suggesting that SOC persistence in these systems is primarily driven by reduced accessibility of SOC through organo‐mineral stabilization, especially in subsoils. In contrast, managed peatlands and topsoils of alpine grasslands exhibited old system ages despite low thermal stability. This suggests that in these soils, SOC persistence is primarily driven by the inhibition of decomposer activity under past anaerobic conditions and low temperatures, rather than by physicochemical stabilization. These findings underscore the sensitivity of SOC dynamics in peatlands and alpine grasslands to changes in environmental conditions. Our study further found evidence of SOC destabilization in croplands and managed peatlands, indicated by low SOC decomposability in combination with long transit times. We propose that the ratio of ^14^C‐based system age to thermal stability (%‐ROC in SOC) could serve as an index of SOC vulnerability, enabling the identification of the dominant mechanisms behind SOC persistence and providing a powerful framework to assess the sensitivity of SOC to climate change and management disturbances.

## Author Contributions

**Luisa I. Minich:** conceptualization, investigation, methodology, writing – original draft, writing – review and editing, visualization, formal analysis, data curation, project administration. **Jeffrey Beem‐Miller:** methodology, formal analysis, writing – review and editing. **Benedict V. A. Mittelbach:** methodology, writing – review and editing. **Dylan Geissbühler:** methodology, writing – review and editing. **Annegret Udke:** methodology, writing – review and editing. **Daniel Wasner:** methodology, writing – review and editing. **Margaux Moreno Duborgel:** methodology, writing – review and editing. **Ciriaco McMackin:** methodology, writing – review and editing. **Alexander S. Brunmayr:** methodology, writing – review and editing. **Lukas Wacker:** methodology. **Philip Gautschi:** methodology. **Negar Haghipour:** methodology. **Markus Egli:** methodology, writing – review and editing. **Jens Leifeld:** methodology, writing – review and editing. **Timothy I. Eglinton:** supervision, funding acquisition, resources, writing – review and editing. **Frank Hagedorn:** funding acquisition, supervision, resources, writing – review and editing, conceptualization, writing – original draft.

## Funding

This work was supported by Schweizerischer Nationalfonds zur Förderung der Wissenschaftlichen Forschung, 193770.

## Conflicts of Interest

The authors declare no conflicts of interest.

## Supporting information


**Appendix S1:** Supporting Information.

## Data Availability

All data presented in this study, as well as the R scripts and input datasets used for modelling system age and transit time, are available in the open‐access Zenodo repository under DOI: https://doi.org/10.5281/zenodo.18019584.
