# Supplementary material for: A Radiocarbon‐Based Framework to Assess Soil Organic Carbon Persistence and Vulnerability Across Land‐Use Types
Source: Glob Chang Biol. 2026 Mar 17;32(3):e70799. doi: 10.1111/gcb.70799 (PMC12993801; doi:10.1111/gcb.70799)
Supplement: Supplementary file 1 — Appendix S1: Supporting Information. [file GCB-32-e70799-s001.docx]

**SUPPORTING INFORMATION**

**A radiocarbon-based framework to assess soil organic carbon persistence and vulnerability across land-use types**

Luisa I. Minich, Jeffrey Beem-Miller, Benedict Mittelbach, Dylan Geissbühler, Annegret Udke, Daniel Wasner, Margaux Moreno Duborgel, Ciriaco McMackin, Alexander S. Brunmayr, Lukas Wacker, Philip Gautschi, Negar Haghipour, Markus Egli, Jens Leifeld, Timothy I. Eglinton, Frank Hagedorn

* Correspondence author: E-mail: luisa.minich@gmail.com

This file includes Appendix S1 – S3, Table S1 – Table S11, Figure S1 – S6, Supporting references.

**Appendix S1:** Effect of carbonate rock weathering on isotopic signatures of microbial respiration.

C isotopic signatures of microbial respiration (Δ^14^CO_2_, ẟ^13^CO_2_) at certain sites and depths indicated potential contributions from carbonate weathering (Table S2). This was inferred from ẟ^13^CO_2_ values exceeding those of SOC and/or Δ^14^CO_2_ values suggesting older CO₂ than SOC, which appeared unreasonable. While we estimated potential carbonate contributions (Table S2), accurately correcting the isotopic signatures of microbial respiration was not feasible due to limited information on endmember values.

To estimate carbonate contributions, we applied a mixing model using ẟ^13^C values as proposed by e.g. (Schindlbacher et al., 2015), assuming an endmember ẟ^13^C value of 0 ‰ for carbonate and equal ẟ^13^C values for SOC and respired CO₂ (MR):

$$f_{carbonate}=\frac{ẟ^{13}{CO_{2}}_{MR} - ẟ^{13}C_{SOC}}{ẟ^{13}C_{carbonate} - ẟ^{13}C_{SOC}}$$

However, this approach was unsuitable for croplands and peatlands due to potential C₄ plant influences, as C₄ plants exhibit ẟ^13^C values between -9 and -17 ‰ (Krüger et al., 2024). Additionally, ẟ^13^CO_2_ values may have been affected by atmospheric air contamination in Exetainer® vials, though triplicate sampling for ẟ^13^C analysis yielded a standard deviation of < 1 ‰ in most cases, suggesting minimal impact.

Given the limitations of ẟ^13^C-based estimates, we also estimated a minimum carbonate contribution using Δ^14^C values for cases where CO₂ appeared older than SOC. Assuming an endmember Δ^14^C value of -1000 ‰ for carbonate and equal Δ^14^C values for SOC and respired CO₂, we calculated:

$$f_{carbonate}=\frac{\Delta^{14}{CO_{2}}_{HR} - \Delta^{14}C_{SOC}}{10}\div100$$

where a 1% carbonate contribution corresponds to a -10 ‰ shift in Δ^14^CO_2_. However, the assumption that SOC-derived CO₂ has the same Δ^14^C value as SOC is likely incorrect, as SOC in soil is not homogeneous. Due to the lack of isotopic data for respired SOC fractions, we estimated potential Δ^14^CO_2_ values using Δ^14^C-to-SOC ratios from sites of the same land-use type which were unaffected by carbonate weathering. The difference between measured and estimated Δ^14^CO_2_ values was then used to apply the equation above.

We derived a corrected Δ^14^CO_2_ value (MR_corr) by averaging carbonate contributions from the three approaches as (Table S3):

$$\Delta^{14}{CO_{2}}_{{MR}_{corr}}=\frac{\Delta^{14}{CO_{2}}_{MR} - \Delta^{14}C_{carbonate} \times f_{carbonate}}{{1-f}_{carbonate}}$$

In some cases, the corrected Δ^14^CO_2_ values appeared unrealistically high, possibly due to overestimated carbonate contributions or the assumed -1000 ‰ endmember value. Carbonate weathering in soils involves reactions with soil water and CO₂ from soil respiration or atmospheric diffusion, forming bicarbonate (HCO₃⁻):

$${CaCO}_{3}\left( calcite \right)+{CO}_{2}+H_{2}O \to{Ca}^{2+}+{2HCO}_{3}^{-}$$

Bicarbonate in soil solution contains both carbonate-derived C (Δ^14^C ~ -1000 ‰) and soil-respired or atmospheric CO₂ (Δ^14^C > -1000 ‰). It can dissociate, precipitate as CaCO₃, or degas as CO₂, contributing abiotically to total soil CO₂ flux (Schindlbacher et al., 2015). Since the measured CO₂ likely included some carbonate-derived CO₂ degassing from solution, its Δ¹⁴C value was probably less depleted than -1000 ‰. For cases with unrealistically high corrected Δ^14^CO_2_ values, we used an alternative HCO₃⁻ endmember of -500 ‰, assuming a 1:1 ratio of carbonate-derived C (-1000 ‰) and soil-derived C (~0 ‰) in the above equation.

**Appendix S2:** Adjustment of bomb curve data sets for each site and depth increment to atmospheric background ^14^CO_2_ and turnover of C inputs.

^14^C values of C inputs were derived from the bomb curve data set for the Northern Hemisphere Zone 1 (Hua et al., 2022). Since the samples were taken in 2022 and 2023, we extended the bomb curve with Δ^14^CO_2_ values measured at Jungfraujoch, Switzerland by ICOS between 2022 to 2023 (Emmenegger et al., 2024) and in 2024 with data provided by Geissbühler (pers. comm.). For sites sampled in 2023, atmospheric background samples were collected and analysed for ^14^CO_2_ in summer 2023 and March 2024 as described in Minich et al., 2025. These measurements revealed a higher dilution of atmospheric ^14^CO_2_ than measured at Jungfraujoch for some sites, likely because of their proximity to fossil C sources (i.e. urban areas, highways). To account for the greater dilution of atmospheric ^14^CO_2_ observed at our sites compared to the Jungfraujoch station, we adjusted the bomb curve for those sites by subtracting the mean seasonal difference (summer and winter) between our site-specific observations and the original bomb curve data, assuming a consistent offset from 1951 to 2024.

Bomb curve data sets were further adjusted to time-lags induced by C inputs where time-lags correspond to the turnover of C inputs. Turnover of C inputs were estimated for each land-use type and depths. For croplands and managed peatlands, we assumed a time-lag of 0.5 years, as they are characterized by annually regrowing plants and seasonal crop rotation. For grasslands and forests, we used literature-based values. Time-lags were adjusted to depth increments or reduced in cases when the modelling did not converge with the ^14^C data with the initially assumed longer time-lag. Land-use type and depth specific time-lags and corresponding literature are presented in Table S3. We assumed that C input in depths > 5 cm originates entirely from belowground sources. If information was available, we adjusted time-lags of organic layer and 0-10 cm of the mineral soil according to the proportion of aboveground and belowground C inputs. It should be noted, that implemented time-lags are solely based on ages of aboveground litter and belowground fine root input while neglecting C inputs from older coarse roots and dissolved organic carbon (DOC) which is especially relevant in subsoil layers. Thus, transit times and system ages from subsoil layers may be overestimated, as longer time-lags would reduce the apparent age of respired CO₂ and SOC. Since transit time is generally smaller than system age, the effect is more pronounced for transit times, given that the ratio of time-lag to transit time is greater than the ratio of time-lag to system age. Figure S6 presents hypothetical time-lags of 45 and 75 years in the depth layer > 40 cm for selected sites and corresponding transit times and system ages.

Assuming a homogenous pool at steady-state, we used the estimated turnover times τ of the input (Table S3) in a simple one-pool model in SoilR (version 1.2.107, Sierra et al., 2012, 2014) defined as:

$$\frac{d^{14}\boldsymbol{C}}{dt}=\boldsymbol{I}_{atm}+\boldsymbol{k}\times\text{14}\boldsymbol{C}- \lambda\text{14}\boldsymbol{C}$$

where *k* is a decomposition rate constant and defined as the inverse of the turnover time (*k* = 1/τ) (Sierra et al., 2014, 2017). Bomb curves were modelled for each site and depth layer and used in the two-pool model for estimating transit time and system age distributions.

**Appendix S3:** Probability distribution function for transit times (TT) and system ages (SA).

The probability distribution functions for transit times (TT) and system ages (SA) of SOC are given by

$f\left( TT \right)={-1}^{T}\times A \times e^{TT \times A}\times\frac{I}{\sum I}$ and $f\left( SA \right)={-1}^{T}\times A \times e^{SA \times A}\times\frac{C}{\sum C}$

where *TT* and *SA* are random variables, *T* is the transpose operator, *A* is the model matrix, *I* is the vector of inputs, *∑I* is the total sum of inputs, and *∑C* is the sum of SOC stocks in all model pools at steady state (Metzler et al., 2018). These probability distribution functions allow for the characterization of the full age distribution of both particles in the system (SA), as well as the output flux (TT). This characterization is particularly useful for understanding SOC dynamics given the tendency for these distributions to deviate from Gaussian normal.

**Table S1:** Site specific information on location, climate, pH, and soil type.

**Table S2:** Estimated contribution of carbonate rock weathering to the isotopic signature of heterotrophically respired CO_2_. Carbonate-corrected Δ^14^CO_2_ values were estimated using a mass-balance approach and assuming isotopic signatures for carbonate of -1000/-500 ‰ for Δ^14^C and of 0 ‰ for δ^13^C. If potential carbonate contribution was > 5%, values and modelled transit times were excluded from analysis (indicated in red).

**Table S3:** Assumed time-lags of carbon inputs for different land-use types, sites, and soil depths. Used in the two-pool modelling of transit time and system age.

| **Site** | **Depth [cm]** | **Land-use type** | **Time-lag** | **References** | **Comment** |
| --- | --- | --- | --- | --- | --- |
| All sites | 0-5 | Temperate grassland | 0.5 | Solly et al., 2013 | adjusted, because 2.0 years was too long |
|  | > 5 |  | 2.0 |  |  |
| Flüelapass, Büelenhorn, Radönt | 0-10 | Alpine grassland | 7.1 | Hitz et al., 2001; Leifeld et al., 2015 | calculated from above- and belowground inputs |
|  | > 10 |  | 8.5 |  |  |
| Schwarzhorn | 0-10 |  | 9.0 |  | calculated from above- and belowground inputs |
|  | > 10 |  | 11.5 |  |  |
| All sites | L (organic layer) | Forest | 0.5 | Solly et al., 2013; Solly et al., 2018 | adjusted |
| Beatenberg | F (organic layer) |  | 3.4 |  | calculated by assuming a ratio of 2:1 for litter:roots |
|  | H (organic layer) |  | 6.4 |  | calculated by assuming a ratio of 1:1 for F:roots |
|  | mineral soil |  | 9.3 |  |  |
| Hölstein | 0-5 |  | 5.9 |  | calculated by assuming a ratio of 1:2 for litter:roots |
|  | > 5 |  | 9.0 |  |  |
| Pfynwald | F (organic layer) |  | 3.0 |  | calculated by assuming a ratio of 2:1 for litter:roots |
|  | mineral soil |  | 9.0 |  |  |
| Lausanne | F (organic layer) |  | 1.6 |  | calculated mean of forest sites with litter layer |
|  | 0-5 |  | 8.6 |  | calculated by assuming a ratio of 1:2 for litter:roots |
|  | > 5 |  | 12.7 |  |  |
| Novaggio | F (organic layer) |  | 1.6 |  | calculated mean of forest sites with litter layer |
|  | 0-5 |  | 7.8 |  | calculated by assuming a ratio of 1:2 for litter:roots |
|  | > 5 |  | 11.3 |  |  |
| Vordemwald | F (organic layer) |  | 1.6 |  | calculated mean of forest sites with litter layer |
|  | 0-5 |  | 7.8 |  | calculated by assuming a ratio of 1:2 for litter:roots |
|  | > 5 |  | 11.3 |  |  |
| All sites | All depths | Cropland | 0.5 |  | assumed |
| All sites | All depths | Managed peatland | 0.5 |  | assumed |

**Table S4:** Monthly mean temperatures for each site.

**Table S5:** Loadings of principle components of PCA analysis. PC1 and PC2 were retained.

**Table S6:** Model output of ANOVA analysis investigating the difference between transit times (TT) and system ages (SA) for each land-use type. Soil depth was included as random effect in the linear mixed effect (LME) model to account for depth-related differences in transit times and system ages.

**Table S7:** Model output of ANOVA analysis investigating the variance between Δ^14^C values of respired CO_2_ and bulk SOC, their modelled transit times (TT), and system ages (SA), respectively, as well as between soil physiochemical properties across land-use types and soil depths. Study sites were included as random effects in the linear mixed effect (LME) model to account for site-specific differences such as soil type.

**Table S8:** Model output of ANOVA analysis investigating the variance between Δ^14^C values of respired CO_2_ and bulk SOC, their modelled transit times (TT), and system ages (SA), respectively, as well as between soil physiochemical properties across land-use types for each soil depth separately. Study sites were included as random effects in the linear mixed effect (LME) model to account for site-specific differences such as soil type.

**Table S9:** Model output of ANOVA analysis investigating the variance between Δ^14^C values of respired CO_2_ and bulk SOC, their modelled transit times (TT), and system ages (SA), respectively, as well as between soil physiochemical properties across soil depths for each land-use type. Study sites were included as random effects in the linear mixed effect (LME) model to account for site-specific differences such as soil type.

**Table S10:** Model output of linear regression (LM) model investigating the relationship between transit time (TT) as well as system age (SA) and soil depth as well as SOC characteristics. The models were performed for each land-use type separately.

**Table S11:** Model output of linear regression (LM) model investigating the relationship between transit time (TT) as well as (SA) and mean annual temperature (MAT) for all grassland sites (including temperate and alpine sites).

**Table S12:** Soil properties across land-use types and soil depths.

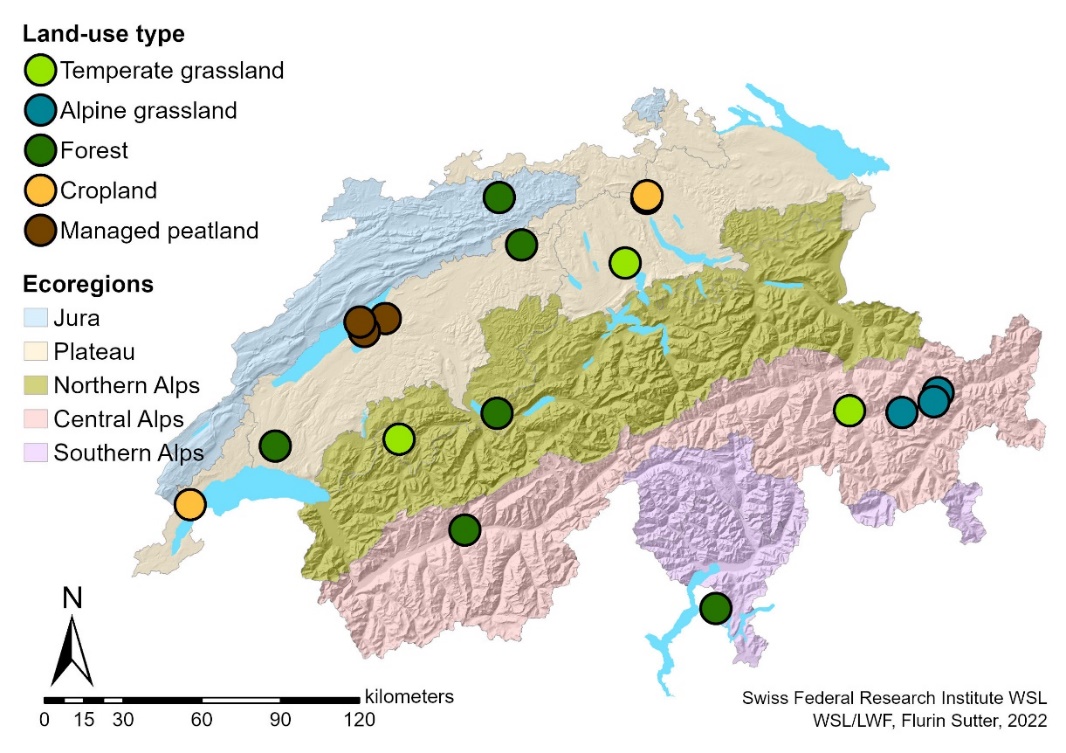


**Figure S1:** Swiss ecoregions and locations of sampling sites for respective land-use types.


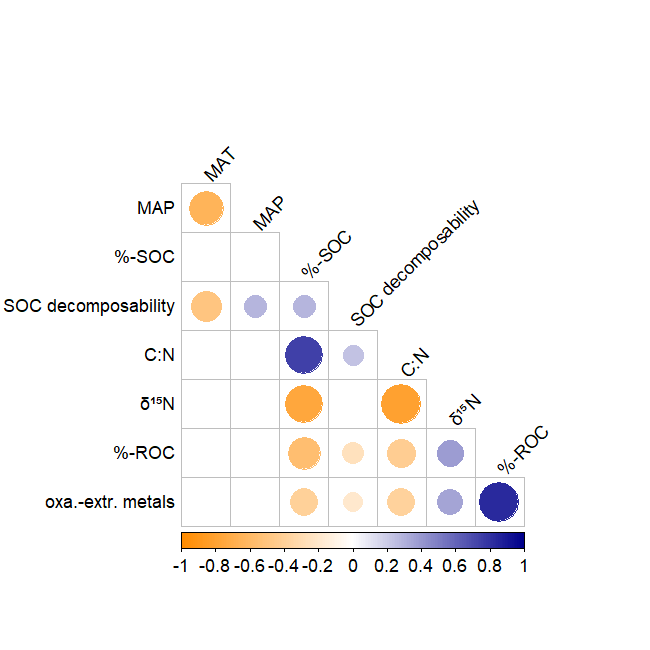

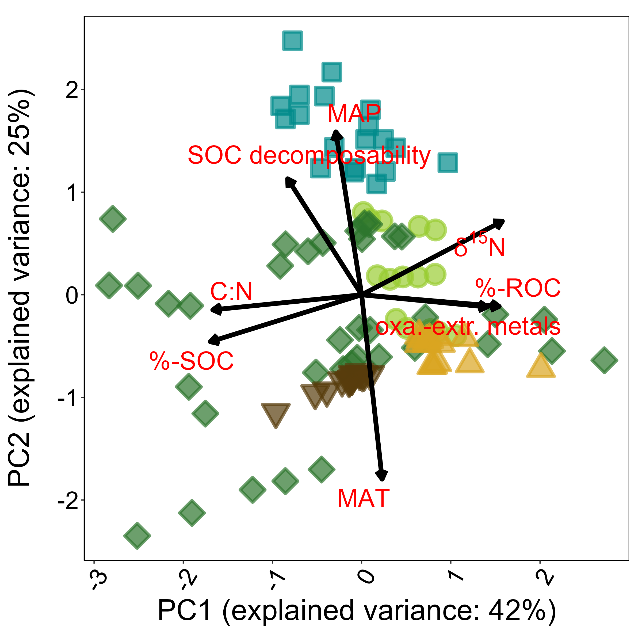

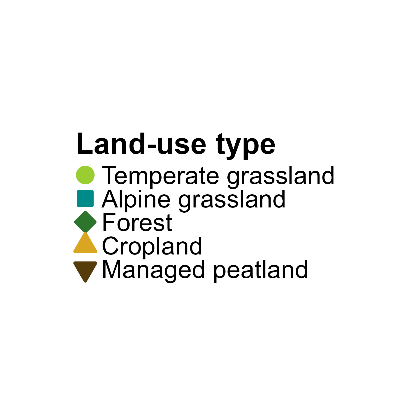


**Figure S2:** **Left:** Results of Principal Component Analysis. Loadings are presented in Table S5. **Right:** Correlation matrix of climatic variables, SOC characteristics, and soil physicochemical properties. Circle size and colour indicate the strength and direction of the relationship (Pearson correlation coefficient) of significant correlations (p < 0.05).


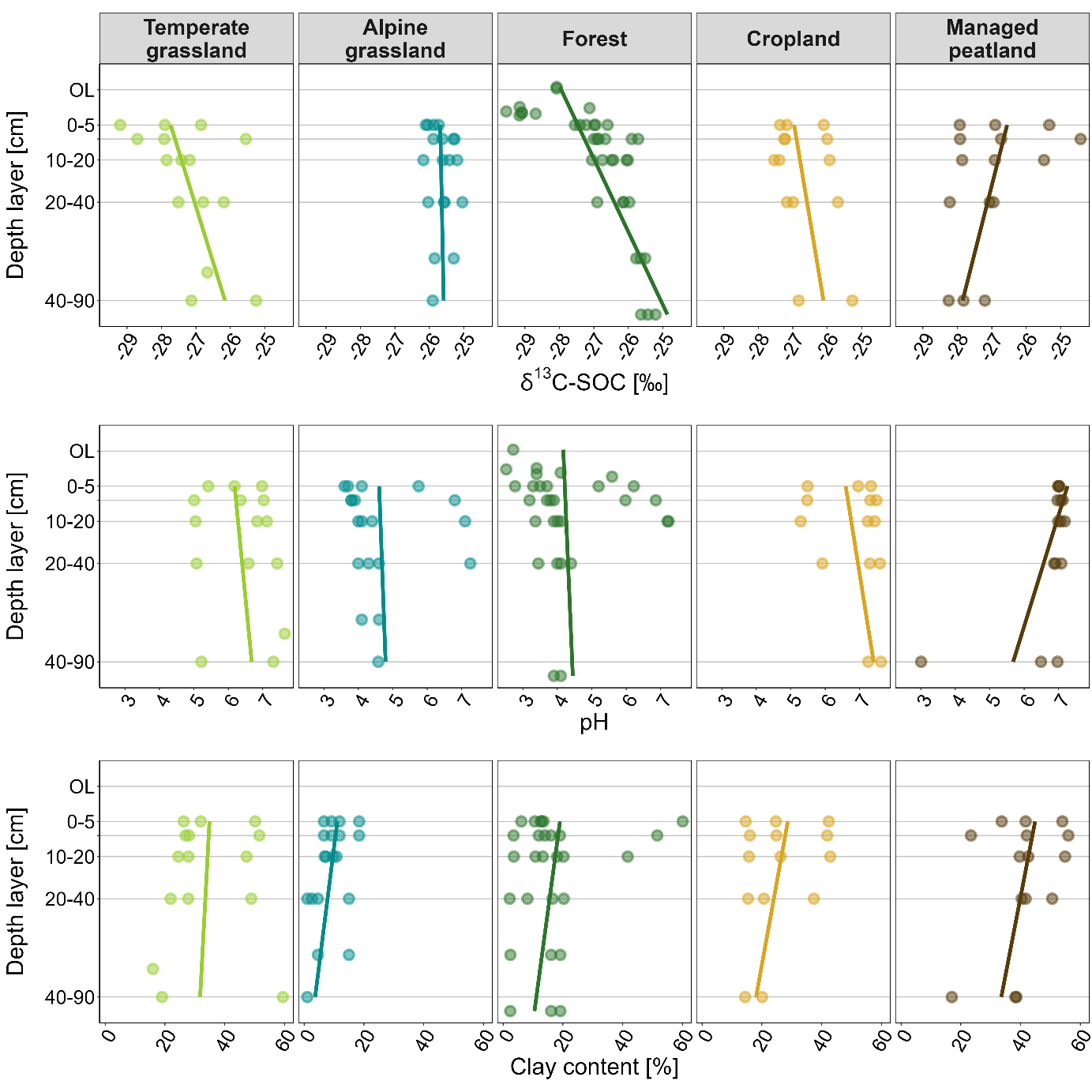


**Figure S3:** δ^13^C of SOC, pH and clay content across land-use types and soil depths.


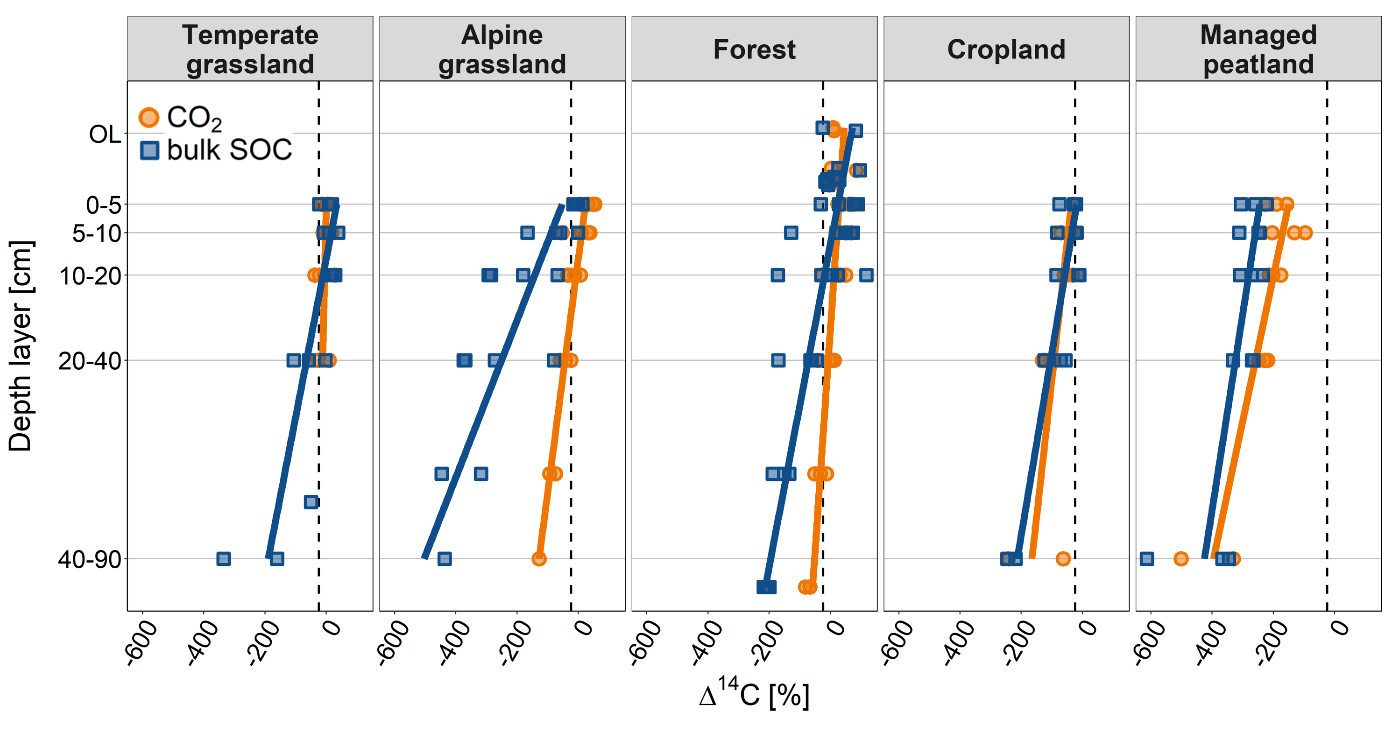


**Figure S4:** Δ^14^C values of microbially respired CO_2_ and in bulk SOC across soil depth for each land-use type. Lines along the depth profiles serve as visual guides to indicate trends. Black dashed lines indicate the mean atmospheric background Δ^14^CO_2_ value (-23.8 ‰) across all land-use types and two seasons (summer, winter) in the year of sampling (2023).


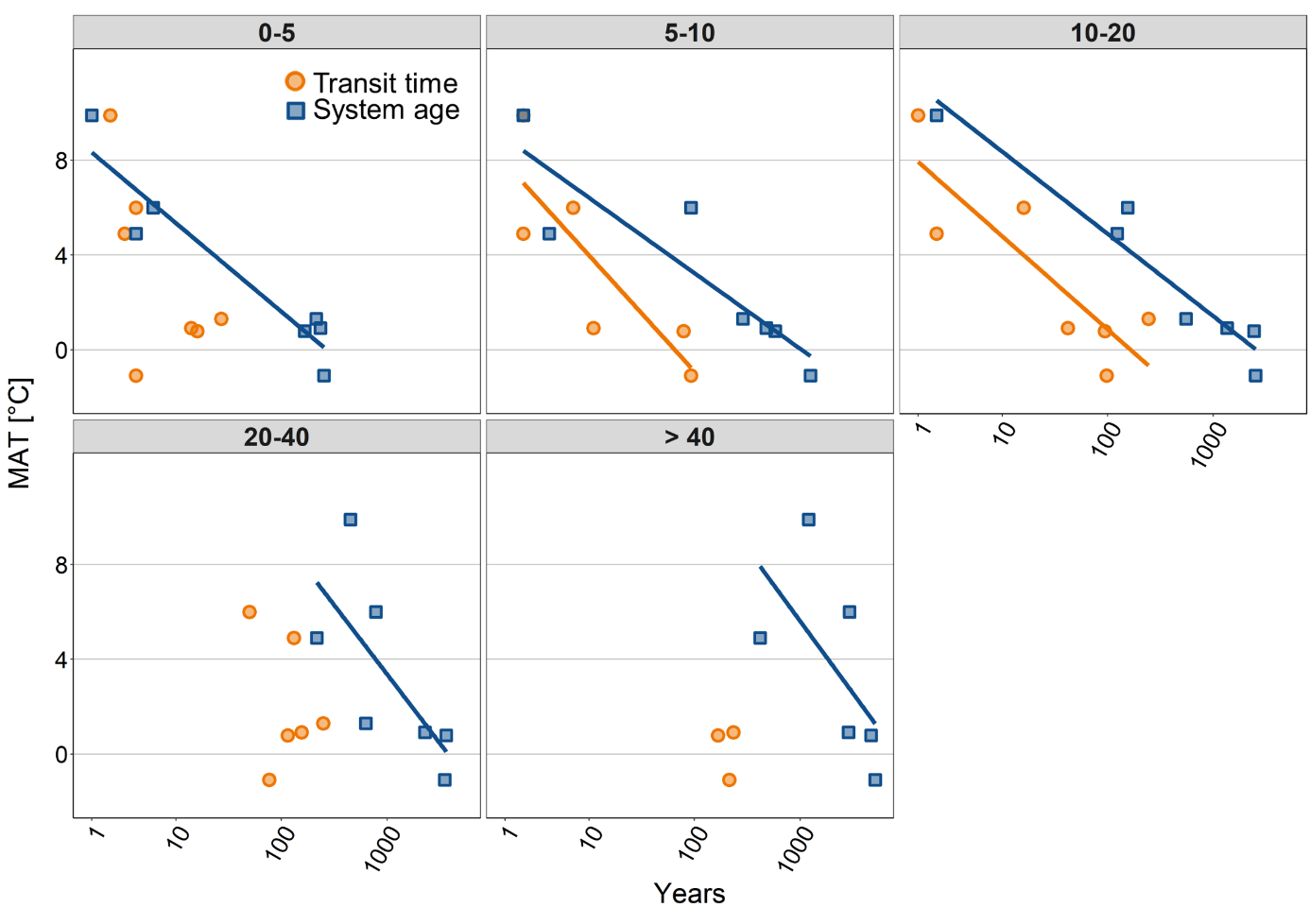


**Figure S5:** Linear relationships between transit times as well as system ages across grasslands sites (including temperate and alpine) in different soil depths. Regression lines are only plotted for significant relationships. Corresponding p-values, R^2^, and coefficients are provided in Table S11.


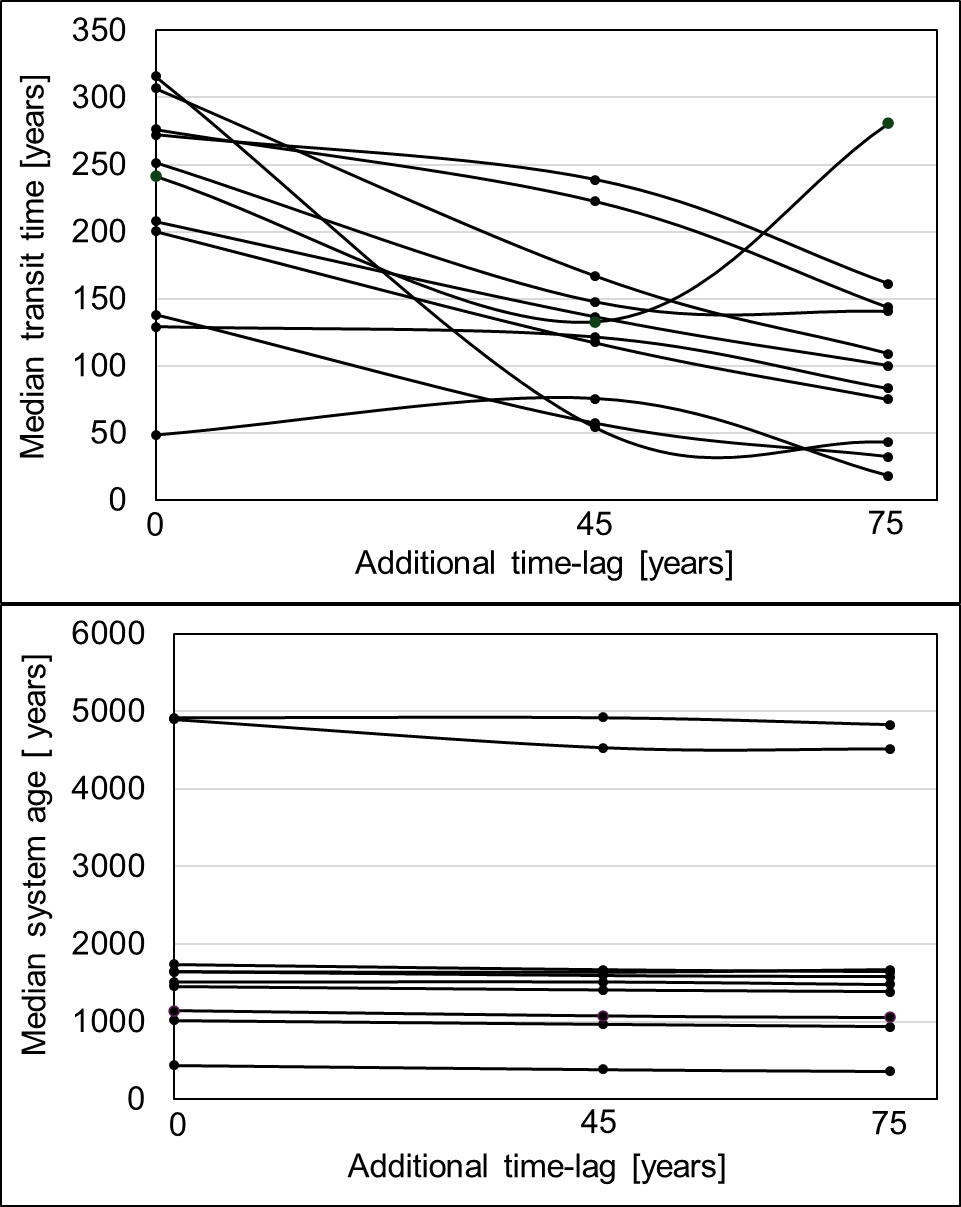


**Figure S6:** Modelled median transit times and system ages assuming an additional time-lag of 45 and 75 years for a subset of sites and in soil depth > 40cm.

**Supporting references**

Emmenegger, L., Leuenberger, M., & Steinbacher, M. (2024). *ICOS ATC 14C Release analysed by ICOS CRL, Jungfraujoch (6.0 m), 2015-09-21–2023-10-02* [Data set]. ICOS RI. https://hdl.handle.net/11676/6c_RZ7NHc2dnZv7d84BMY_YY

Hitz, C., Egli, M., & Fitze, P. (2001). Below-ground and above-ground production of vegetational organic matter along a climosequence in alpine grasslands. *Journal of Plant Nutrition and Soil Science*, *164*(4), 389. https://doi.org/10.1002/1522-2624(200108)164:4%253C389::AID-JPLN389%253E3.0.CO;2-A

Hua, Q., Turnbull, J. C., Santos, G. M., Rakowski, A. Z., Ancapichún, S., De Pol-Holz, R., Hammer, S., Lehman, S. J., Levin, I., Miller, J. B., Palmer, J. G., & Turney, C. S. M. (2022). ATMOSPHERIC RADIOCARBON FOR THE PERIOD 1950–2019. *Radiocarbon*, *64*(4), 723–745. https://doi.org/10.1017/RDC.2021.95

Krüger, N., Finn, D. R., & Don, A. (2024). Soil depth gradients of organic carbon-13 – A review on drivers and processes. *Plant and Soil*, *495*(1–2), 113–136. https://doi.org/10.1007/s11104-023-06328-5

Leifeld, J., Meyer, S., Budge, K., Sebastia, M. T., Zimmermann, M., & Fuhrer, J. (2015). Turnover of Grassland Roots in Mountain Ecosystems Revealed by Their Radiocarbon Signature: Role of Temperature and Management. *PLOS ONE*, *10*(3), e0119184. https://doi.org/10.1371/journal.pone.0119184

Metzler, H., Müller, M., & Sierra, C. A. (2018). Transit-time and age distributions for nonlinear time-dependent compartmental systems. *Proceedings of the National Academy of Sciences*, *115*(6), 1150–1155. https://doi.org/10.1073/pnas.1705296115

Minich, L. I., Geissbühler, D., Tobler, S., Udke, A., Brunmayr, A. S., Moreno Duborgel, M., McMackin, C., Wacker, L., Gautschi, P., Haghipour, N., Egli, M., Kahmen, A., Leifeld, J., Eglinton, T. I., & Hagedorn, F. (2025). *Conceptualising carbon cycling pathways across different land-use types based on rates and ages of soil-respired CO_2_*. Copernicus GmbH. https://doi.org/10.5194/egusphere-2025-2267

Schindlbacher, A., Borken, W., Djukic, I., Brandstätter, C., Spötl, C., & Wanek, W. (2015). Contribution of carbonate weathering to the CO2 efflux from temperate forest soils. *Biogeochemistry*, *124*(1–3), 273–290. https://doi.org/10.1007/s10533-015-0097-0

Sierra, C. A., Müller, M., Metzler, H., Manzoni, S., & Trumbore, S. E. (2017). The muddle of ages, turnover, transit, and residence times in the carbon cycle. *Global Change Biology*, *23*(5), 1763–1773. https://doi.org/10.1111/gcb.13556

Sierra, C. A., Müller, M., & Trumbore, S. E. (2014). Modeling radiocarbon dynamics in soils: SoilR version 1.1. *Geoscientific Model Development*, *7*(5), 1919–1931. https://doi.org/10.5194/gmd-7-1919-2014

Solly, E. F., Brunner, I., Helmisaari, H.-S., Herzog, C., Leppälammi-Kujansuu, J., Schöning, I., Schrumpf, M., Schweingruber, F. H., Trumbore, S. E., & Hagedorn, F. (2018). Unravelling the age of fine roots of temperate and boreal forests. *Nature Communications*, *9*(1), 3006. https://doi.org/10.1038/s41467-018-05460-6

Solly, E., Schöning, I., Boch, S., Müller, J., Socher, S. A., Trumbore, S. E., & Schrumpf, M. (2013). Mean age of carbon in fine roots from temperate forests and grasslands with different management. *Biogeosciences*, *10*(7), 4833–4843. https://doi.org/10.5194/bg-10-4833-2013
